# Phase I/II, open-label, multicenter study of durvalumab in combination with tremelimumab in pediatric patients with advanced solid tumors

**DOI:** 10.3389/fonc.2026.1680081

**Published:** 2026-05-15

**Authors:** Darren Hargrave, Lynley V. Marshall, Nicolas André, Julie Krystal, Brian H. Ladle, Karen A. Robbins, Stephan Hois, Jon Armstrong, Sarah Donegan

**Affiliations:** 1 University College London Great Ormond Street Institute of Child Health and Great Ormond Street Hospital for Children, London, United Kingdom; 2 Pediatric and Adolescent Oncology Drug Development Unit, Oak Centre for Children and Young People, The Royal Marsden Hospital and Division of Clinical Studies, The Institute of Cancer Research, London, United Kingdom; 3 Pediatric Hematology and Oncology Department, Hôpital pour Enfant de La Timone, Assistance Publique - Hôpitaux de Marseille (AP-HM), and Reverse Molecular Pharmacology in Pediatric Oncology, Centre de Recherche en Cancérologie de Marseille (CRCM), Aix-Marseille Université, CNRS, INSERM, Marseille, France; 4 Zucker Hofstra School of Medicine, Department of Pediatrics, Cohen Children’s Medical Center, New Hyde Park, NY, United States; 5 Sidney Kimmel Comprehensive Cancer Center, Johns Hopkins University School of Medicine, Baltimore, MD, United States; 6 Global Patient Safety, Oncology, AstraZeneca, Gaithersburg, MD, United States; 7 Late-stage Development Oncology R&D, AstraZeneca, Gaithersburg, MD, United States; 8 Oncology Biometrics, AstraZeneca, Cambridge, United Kingdom; 9 Oncology R&D, AstraZeneca, Gaithersburg, MD, United States

**Keywords:** antitumor activity, biomarker analysis, combination therapy, cytotoxic T-lymphocyte associated protein 4 inhibitor, pharmacokinetics, programmed death ligand 1 inhibitor, solid tumors

## Abstract

**Purpose:**

We conducted a phase I/II study to evaluate durvalumab (D; anti-PD-L1) in combination with tremelimumab (T; anti–cytotoxic T-lymphocyte antigen 4) in pediatric patients with relapsed/refractory solid tumors.

**Patients and methods:**

The dose-finding phase (phase I) assessed the safety and pharmacokinetics of D+T at two dose levels to determine the recommended phase II dose (RP2D). The primary endpoint for the dose-expansion phase (phase II) was antitumor activity (by Response Evaluation Criteria in Solid Tumors v1.1) at the RP2D. Assessment of circulating cells reflecting immune cell activation was a secondary endpoint.

**Results:**

Based on safety and pharmacokinetic data in the dose-finding phase (n = 29), the adult-equivalent RP2D was determined as D (30 mg/kg) + T (1 mg/kg). In the dose-expansion phase, 21 patients (sarcoma, n = 11; solid tumors, n = 10) were treated at the RP2D. Of patients with sarcoma, none achieved an objective response and one had stable disease ≥7 weeks. Of the patients with solid tumors, one (chordoma) had a confirmed partial response (10.8 months duration) and one (renal cell carcinoma) had stable disease ≥7 weeks. Treatment-related adverse events occurred in 76% of patients (19% as grade 3 or 4). No adverse events leading to death were reported. Across both phases treatment with D+T resulted in an increase in CD4+Ki67+ T cells.

**Conclusions:**

D (30 mg/kg) + T (1 mg/kg) had limited antitumor activity in this pediatric population; however, the safety profile was manageable and consistent with the known safety profile in adult patients, with no new safety concerns identified.

**Clinical trial registration:**

ClinicalTrials.gov, identifier NCT03837899; EudraCT, identifier 2018-003118-42.

## 1 Introduction

Multiagent chemotherapy and/or radiotherapy have improved survival outcomes in children with solid tumors; however, more than 50% of children surviving cancer develop chronic health conditions later in life due to long-term effects of cancer therapy (1). Despite current standard-of-care treatment regimens, children with relapsed/refractory solid tumors have poor survival outcomes (2). By targeting the immune system and enhancing antitumor immunity, immunotherapies have the potential to improve outcomes in children with solid tumors (3), with the avoidance of long-term adverse health effects of chemotherapy and radiotherapy, thereby improving their quality of life.

Based on improved outcomes demonstrated with immune checkpoint inhibitors (ICIs) in adult patients across a variety of cancer types (4), ICI therapies have been investigated in children with solid tumors and lymphoma, including those with relapsed/refractory disease (5–11). Several phase I/II studies of ICI monotherapies (ipilimumab, nivolumab, pembrolizumab, atezolizumab, avelumab) and combination regimens (nivolumab plus ipilimumab) have been conducted in pediatric populations, but limited antitumor activity has been observed, with objective response rates ranging from 0% to 5.9% for children with a solid tumor, including sarcoma.

Durvalumab is a selective, high-affinity human immunoglobulin G1 (IgG1) kappa light chain monoclonal antibody that binds programmed death ligand 1 (PD-L1) to block the interaction of PD-L1 with programmed cell death 1 (PD-1) and cluster of differentiation 80 (CD80) to overcome inhibition of T cell activation. Tremelimumab is an IgG2 monoclonal antibody that selectively binds to cytotoxic T-lymphocyte antigen 4 (CTLA-4), thereby blocking its interactions with the B7 ligands (CD80 and CD86) and allowing binding of the B7 ligands with CD28 to enhance T cell activation. Because of the complementary mechanisms of action of durvalumab and tremelimumab, the combination of both agents may enable antitumor T cell responses that are not achieved with either agent alone (12, 13). In phase III trials, durvalumab plus tremelimumab administered with chemotherapy has demonstrated improved progression-free survival (PFS) and overall survival (OS) compared with chemotherapy alone in adults with metastatic non–small-cell lung cancer (NSCLC) (13) and durvalumab plus tremelimumab has demonstrated improved OS compared with sorafenib alone in adults with unresectable hepatocellular cancer (14). However, the efficacy and safety of the durvalumab and tremelimumab combination is unknown in pediatric cancers. A phase I/II study of this dual ICI combination was conducted in children with relapsed/refractory solid tumors.

An earlier study evaluated 115 pediatric tumor samples (at diagnosis) for PD-L1 expression and the presence of CD8+ tumor-infiltrating lymphocytes (15). PD-L1 positivity was defined as >5% membrane staining and included the following tumors (in order of PD-L1 expression): alveolar rhabdomyosarcoma (86%), high-risk neuroblastoma (72%), Ewing sarcoma (57%), and osteosarcoma (50%). The results of the tumor microenvironment analysis were correlated with clinical outcomes and the findings from this study demonstrated that patients with tumors that were characterized as PD-L1+ high and CD8+ positive had significantly higher overall survival, suggesting that patients with this phenotype could potentially benefit from PD-1/PD-L1 blockade. Supported by the preclinical data, initial pediatric studies evaluated PD-1/PD-L1 inhibitor monotherapy and subsequently in-order to exploit the complementary action of T-cell activation from an anti-CTLA-4 approach, combination studies have been conducted including the present study and the prior ADVL 1412 study. At the time of study conception, the rationale for testing the PD-L1/CTLA-4 combination, as with the nivolumab/ipilimumab regimen evaluated in the ADVL 1412 study, was based on responses observed in adult cancer patients receiving a dual checkpoint regimen, including patients with low PD-L1 expression. Additionally, the correlation between PD-L1 expression and response had not been formally evaluated in pediatric tumors. This study is the first clinical study to expand beyond the PD-L1 biomarker characterization, to also characterize the immune response profile following the administration of a dual checkpoint inhibitor regimen in a relapsed/refractory setting. The study design included 4 disease cohorts in the dose-finding phase as neuroblastoma, sarcoma (bone) and sarcoma (soft-tissue), and any solid tumor; a broad selection of relapsed/refractory tumors (excluding primary CNS tumors), regardless of PD-L1 expression, was selected to offer patients' exposure to a dual ICI regimen with the possibility of an immune response linked to efficacy due to inhibition of these two checkpoint pathways.

## 2 Patients and methods

### 2.1 Study design and patients

This was an open-label, international, multicenter phase I/II study in pediatric patients with relapsed or refractory malignant solid tumors. The study comprised two sequential phases: dose finding and dose expansion. Patients were enrolled at 19 sites in seven countries from March 2019 through May 2022.

#### 2.1.1 Dose-finding phase

A minimum of 12 evaluable patients were required for the dose-finding phase; patients were allocated to one of two arms for each of the dose levels based on patient weight (≥35 kg and <35 kg). The dose escalation plan followed a modified 3 + 3 design taking into consideration the pharmacokinetic (PK) exposure (i.e., achievement of equivalent adult exposure) and presence of a dose-limiting toxicity (DLT). Dose escalation decisions and determination of the RP2D to be explored in the dose expansion phase, were based on safety and PK data reviewed by the Data Review Committee (DRC) comprised of study investigators and the Sponsor. Patients were enrolled into one of the following disease cohorts: neuroblastoma, bone sarcomas (osteosarcoma, Ewing’s sarcoma), soft-tissue sarcomas (rhabdomyosarcoma, non-rhabdomyosarcoma soft-tissue sarcoma, and other sarcomas), and solid tumors.

#### 2.1.2 Dose-expansion phase

Patients were enrolled into one of the following disease cohorts: sarcoma (bone sarcomas and soft-tissue sarcomas) and solid tumors. For the sarcoma cohort at least 40% were required to include a soft-tissue sarcoma subtype. For this cohort, 11 patients were initially enrolled in the first stage; the Simon-2 stage design was applied to allow a provision to include expansion cohort (i.e., an additional 15 patients), based on protocol-defined response criteria. Ten patients were enrolled into the solid tumor cohort; as this was a heterogenous disease cohort, Simon rules were not applicable for this cohort, instead descriptive statistics were used. All patients, regardless of weight, received the RP2D regimen.

Eligibility requirements for all phases included: age from birth to <18 years, no prior exposure to ICIs or genetically engineered cellular therapies, a Lansky play performance scale ≥50 for patients aged ≥1 to <16 years, and a Karnofsky performance status score ≥50 for those aged ≥16 years, at least one lesion not previously irradiated that could be accurately evaluated or measured by Response Evaluation Criteria in Solid Tumors version 1.1 (RECIST v1.1) (or evaluable disease as assessed using common response methods by disease type), adequate organ and bone marrow function, and a life expectancy of ≥3 months. If a diagnostic tumor tissue sample was available, provision of the sample for evaluation of PD-L1 expression was required. All patients had relapsed or refractory disease.

The study was approved by the institutional review board or ethics committee at each site and the National Ethics Advisory Committee of France and was conducted in accordance with the principles of the Declaration of Helsinki and Good Clinical Practice guidelines. Prior to enrollment, all patients (or their legal representatives) provided written informed consent to participate. Key modifications to the study design are described in the **Supplementary Material**. The study was sponsored by AstraZeneca and is registered with ClinicalTrials.gov (NCT03837899) and EudraCT (Number 2018-003118-42).

### 2.2 Treatment plan

Dose-finding: Durvalumab was administered intravenously as a single dose (cycle 1) followed by the combination of durvalumab plus tremelimumab, administered intravenously every 4 weeks (one treatment cycle) for four cycles (cycles 2–5), and then as durvalumab monotherapy every 4 weeks. Two dose levels were evaluated to determine the recommended phase II dose (RP2D): dose level 1, durvalumab 20 mg/kg and tremelimumab 1 mg/kg (D20+T1), and dose level 2, durvalumab 30 mg/kg and tremelimumab 1 mg/kg (D30+T1). Patients were allocated to a treatment group based on body weight (arm A: ≥35 kg; arm B: <35 kg). In the dose-expansion phase, patients received the RP2D of durvalumab plus tremelimumab starting on cycle 1 for 4 cycles, followed by durvalumab monotherapy.

### 2.3 Primary endpoints

The primary endpoints for the dose-finding phase were (i) the determination the adult-equivalent exposure/maximum tolerated dose/pediatric RP2D of durvalumab plus tremelimumab, and (ii) the safety profile of durvalumab plus tremelimumab, and durvalumab as monotherapy following combination therapy. The primary endpoint for the dose-expansion phase was the preliminary antitumor activity of durvalumab plus tremelimumab (followed by durvalumab monotherapy maintenance), administered at the RP2D. Antitumor activity was assessed by investigator as best overall response and objective response rate (ORR) using RECIST v1.1 criteria. Additional study endpoints included PFS and OS.

### 2.4 Secondary and safety endpoints

Secondary endpoints included determination of PK parameters of both durvalumab and tremelimumab (as a combination regimen) and of durvalumab administered as monotherapy (as a maintenance regimen), in both phases. Additional secondary endpoints included determination of immunogenicity (i.e., the number of patients who developed detectable antidrug antibodies [ADAs]) and flow cytometry of immune cells (natural killer [NK], CD4, CD8 T cells, B cells, and proliferating T cells) in at least 10 enrolled patients; data from the flow cytometry analysis were to be compared with similar data available for adult patients receiving a similar durvalumab and tremelimumab combination (15).

The safety objective was to determine the safety profile and tolerability for patients in the dose-expansion cohort who received four cycles of durvalumab and tremelimumab followed by maintenance durvalumab. Adverse events (AEs) were graded according to the National Cancer Institute Common Terminology Criteria for AEs, Version 5. AEs of special interest were recorded, which included but were not limited to events with a potential inflammatory or immune-mediated mechanism that may have required more frequent monitoring and interventions such as steroids or immunosuppressants. An immune-mediated AE was defined as an AE of special interest associated with drug exposure and consistent with an immune-mediated mechanism of action and for which there was no clear alternate etiology. AEs were assessed up to 90 days after the last dose of durvalumab or tremelimumab or until initiation of subsequent anticancer therapy after discontinuation of study treatment, whichever occurred first.

### 2.5 Immune activation and biomarker analyses

As a secondary endpoint, samples collected from enrolled patients were assessed for the effects of the combination treatment on circulating quantities of T, B, and NK cells (TBNK) and proliferating T cells (Ki67+). This was done using analytically validated flow cytometry assays using antibodies against CD3, CD4, CD8, CD14, CD19, CD56/CD16, and CD45 to enumerate TBNK cells. An additional antibody targeting Ki67 was used to enumerate proliferating T cells, while antibodies to human leukocyte antigen DR (HLA-DR), IgG1/IgG2a, CD278 (inducible co-stimulator; ICOS), CD38, CD45RA, and CD197 (C-C chemokine receptor 7; CCR7) were used to quantify activated and memory T cells. These immune activation profiles were compared with a cohort of adult patients with NSCLC who received the same treatment combination and were tested using the same assays (15).

As an exploratory endpoint, PD-L1 expression was assessed by immunohistochemistry in pretreatment tumor tissue samples using the investigational VENTANA PD-L1 (SP263) Assay (Roche Diagnostics, Tucson, AZ, USA). PD-L1 expression was scored using a tumor area positivity (TAP) score (16). The TAP score was determined by visually aggregating/estimating the area covered by PD-L1–positive tumor cells and tumor-associated immune cells relative to the total tumor area, using a cutoff of 10%. The relationship among PD-L1 expression, tumor type, and clinical outcomes was assessed.

### 2.6 Statistical analyses

There was no formal statistical testing for efficacy data in the dose-finding phase. In the dose-expansion phase, based on the assumption that the ORR for the null hypothesis was 10% in the sarcoma cohort, no additional patients were enrolled if there were fewer than two confirmed responses among the first 11 evaluable patients. Descriptive statistics were used for all variables, as appropriate, with continuous variables summarized by the number of observations, mean, standard deviation, median, minimum, and maximum. Categorical variables were summarized by frequency counts and percentages. The results of all statistical analyses used a 90% confidence interval (CI), unless otherwise stated. ORR was based on the programmatically derived RECIST v1.1 using investigator tumor data, and the point estimate and 90% CI (two-sided) of the ORR were calculated. The CI was calculated using an exact Mid-P method. Analyses of PFS and OS were performed using Kaplan–Meier methods.

## 3 Results

### 3.1 Patients

Patient baseline demographics and disease characteristics are provided in **Table 1**. All enrolled patients were aged ≥1 year and <18 years at baseline; median age was 11.0 (range, 3–17) and 14.0 (range, 1–17) years in the dose-finding and the dose-expansion cohorts, respectively. Overall, 34.0% of patients had received more than three prior therapies and 66.0% of patients had metastatic disease at baseline. The distribution of tumor types across the study was sarcoma (29 [58.0%]), neuroblastoma (3 [6.0%]), and other solid tumors (18 [36.0%]). The study population was considered generally representative of pediatric patients with solid tumors in North America and Western Europe (**Supplementary Table 1**). However, due to the limited enrollment of this phase I/II study, the demographics are not reflective of a global population of pediatric patients with solid tumors, specifically as Black and Asian patients were under-represented in this study.

**Table 1:** Demographic and disease characteristics of the patients at baseline (at time of study enrollment).

Characteristic	Dose-finding phase (n = 29)	Dose-expansion phase (n = 21)	Total (N = 50)
Age, median (range), year	11.0 (3–17)	14.0 (1–17)	11.5 (1–17)
Age group, n (%), year			
≥1 to <6	4 (13.8)	2 (9.5)	6 (12.0)
6 to <12	11 (37.9)	8 (38.1)	19 (38.0)
12 to <18	14 (48.3)	11 (52.4)	25 (50.0)
Sex, n (%)			
Male	14 (48.3)	10 (47.6)	24 (48.0)
Female	15 (51.7)	11 (52.4)	26 (52.0)
Race, n (%)			
Black or African American	3 (10.3)	0	3 (6.0)
American Indian or Alaska Native	0	1 (4.8)	1 (2.0)
Asian	1 (3.4)	2 (9.5)	3 (6.0)
White	19 (65.5)	14 (66.7)	33 (66.0)
Other	6 (20.7)	4 (19.0)	10 (20.0)
Ethnicity, n (%)			
Hispanic or Latinx	5 (17.2)	2 (9.5)	7 (14.0)
Not Hispanic or Latinx	24 (82.8)	19 (90.5)	43 (86.0)
Weight group, n (%), kg			
<35	11 (37.9)	7 (33.3)	18 (36.0)
≥35	18 (62.1)	14 (66.7)	32 (64.0)
Prior systemic anticancer therapies, n (%)			
Cytotoxic chemotherapy	27 (93.1)	19 (90.5)	46 (92.0)
Immunotherapy	6 (20.7)	4 (19.0)	10 (20.0)
Biologic therapy	3 (10.3)	0	3 (6.0)
Experimental therapy	2 (6.9)	2 (9.5)	4 (8.0)
Radiopharmaceuticals	1 (3.4)	0	1 (2.0)
Other*	11 (41.4)	7 (33.3)	18 (36.0)
Number of prior anticancer therapies, n (%)			
0	0	0	0
1	6 (20.7)	5 (23.8)	11 (22.0)
2	4 (13.8)	4 (19.0)	8 (16.0)
3	4 (13.8)	6 (28.6)	10 (20.0)
>3	13 (44.8)	4 (19.0)	17 (34.0)
Unknown	2 (6.9)	2 (9.5)	4 (8.0)
Prior radiotherapy, n (%)	15 (51.7)	16 (76.2)	31 (62.0)
Prior surgery, n (%)	28 (96.6)	16 (76.2)	44 (88.0)
Lansky or Karnofsky performance score, n (%)			
100	12 (41.4)	11 (52.4)	23 (46.0)
90	9 (31.0)	5 (23.8)	14 (28.0)
80	3 (10.3)	3 (14.3)	6 (12.0)
70	4 (13.8)	1 (4.8)	5 (10.0)
60	1 (3.4)	1 (4.8)	2 (4.0)
Time from initial diagnosis, n (%), months			
≤24	15 (51.7)	11 (52.4)	26 (52.0)
>24	14 (48.3)	10 (47.6)	24 (48.0)
Disease classification, n (%)			
Metastatic	18 (62.1)	15 (71.4)	33 (66.0)
Locally advanced	1 (3.4)	2 (9.5)	3 (6.0)
Both	8 (27.6)	3 (14.3)	11 (22.0)
Missing or not applicable	2 (6.9)	1 (4.8)	3 (6.0)
Tumor types, n (%)			
Sarcoma Bone (Osteosarcoma and Ewing sarcoma) Soft-tissue sarcoma Alveolar soft-part sarcoma Clear-cell sarcoma Embroynal rhabdomyosarcoma ETRAN-TR Fibromyxoid sarcoma MPSNT Poorly differentiated chordoma Undifferentiated sarcoma Histology not recorded *	18(62.1) 9 6 1 1 0 1 1 1 1 0 3	11(52.4) 5 6 1 0 2 0 0 1 0 1 0	29 (58.0) 14 (48.2) 12 (41.3) (3 missing histology)
Neuroblastoma	3 (10.3)	0	3 (6.0)
Solid tumors Adrenocortical carcinoma Chordoma (one poorly differentiated) Clear-cell adenocarcinoma/carcinoma Lymphoepithelial carcinoma Hepatocellular carcinoma Malignant rhabdoid tumor Nephroblastoma Renal cell carcinoma Ovarian cystoadenocarcinoma Uveal melanoma UCNT Wilms tumor	8 (27.6) 1 1 0 0 1 1 1 2 0 0 1 0	10 (47.6) 0 1 2 1 0 1 0 1 1 1 0 2	18 (36.0)

* Includes use of tyrosine kinase and mechanistic target of rapamycin (mTOR) inhibitors, a retinoid, and an immunomodulating imide drug (IMiD).

* Reflects instances where tumor type was not properly captured in the database.

ETRAN, Embroynal tumor with multi-layered rosettes; MPNST, Malignant peripheral nerve sheath tumor; UCNT, Undifferentiated nasopharyngal cancer.

In the dose-finding phase, 29 patients received study treatment (**Supplementary Figure 1**). Ten patients were enrolled at dose level 1 (20 mg/kg), of whom nine (90.0%) received both durvalumab and tremelimumab; 19 patients were enrolled at dose level 2 (30 mg/kg), of whom 12 (63.2%) received both durvalumab and tremelimumab. Reason for not progressing to receive tremelimumab at dose-level 1 was disease progression; reasons in dose-level 2 were disease progression (5), withdrawal by parent, and patient lost to follow-up. The actual treatment duration (inclusive of maintenance durvalumab) was a median of 0.92 months (range, 0.9–24.6) at dose level 1 and 0.92 months (range, 0.9–30.1) at dose level 2. In the dose-finding phase, three patients discontinued treatment. One patient discontinued because of withdrawal of consent by their parent/guardian and another patient (osteosarcoma) who had achieved a durable partial response had treatment discontinued because of a planned thoracic resection of a residual mass. The third patient (renal cell carcinoma) who also achieved a durable partial response, was removed from this study due to closure of the study; subsequently this patient was transitioned to a post-treatment access program to continue receiving maintenance durvalumab. As per the post-access program, routine monitoring is performed but not recorded with only collection of reports of serious AEs and AEs of special interest related to treatment and the final disposition of patient.

In the dose-expansion phase, 21 patients received treatment with the RP2D of D30+T1 (11 in the sarcoma cohort; 10 in the solid tumor cohort) (**Supplementary Figure 2**). Two patients discontinued treatment because of an AE (grade 3 pulmonary thrombosis after two cycles of the combination regimen [not treatment related] and grade 3 thrombocytopenia after one cycle of the combination regimen [possibly treatment related]). The median actual treatment durations in the sarcoma and solid tumor cohorts were 1.84 (range, 0.9–3.6) and 1.72 (range, 0.9–3.7) months, respectively.

Disease progression was the most common reason for treatment discontinuation across both study phases. At data cutoff, 24 of 29 (82.8%) and 15 of 21 (71.4%) of patients had died in the dose-finding and dose-expansion phases, respectively, with most deaths (19 of 24 [79.2%] and 8 of 15 [53.3%]) recorded >90 days after the last dose of study treatment.

### 3.2 Pharmacokinetics

Determination of the adult exposure equivalent dose of both durvalumab and tremelimumab was the primary objective of the dose-finding phase. Equivalent adult exposure was declared if the lower limit of the 80% CI of the pediatric exposure was more than 50% of the adult exposure. Using the reference data for the durvalumab 20 mg/kg every 4 weeks regimen, the target area under the curve from time zero to 28 days (AUC _0–28_) was ≥2105 day*µg/mL, reflecting 50% of the adult exposure (4210 day*µg/mL); for tremelimumab, the target AUC _0–28_ was ≥119.5 day*µg/mL. For the PK analysis, 10 and 12 evaluable samples were available, for dose level 1 and dose level 2, respectively.

At the durvalumab dose level 1 (20 mg/kg), target systemic exposure was achieved in 5 of 6 (83%) patients in the ≥35 kg weight group and 2 of 3 (67%) patients in the <35 kg weight group. Target tremelimumab systemic exposure at the 1 mg/kg dose was achieved in 9 (100%) patients and was consistent across both weight groups. All 12 patients who received durvalumab at dose level 2 (30 mg/kg) met or exceeded the target systemic exposure across both weight groups. The mean exposure was higher at 30 mg/kg compared with 20 mg/kg, with a trend toward higher mean concentrations in patients ≥35 kg than in patients <35 kg. For the mean AUC, C_max_, and C_min_, exposure in patients <35 kg was ~70% to 75% of the exposure for patients ≥35 kg (except where C_min_ was approximately half) for both the 20 and 30 mg/kg doses.

Geometric mean serum concentrations for durvalumab and tremelimumab are shown in **Supplementary Figure 3**. The complete summary of PK parameters for durvalumab and tremelimumab in the dose-finding phase are summarized in **Supplementary Tables 4**, **5**, respectively. Based on the PK results and safety data from the dose-finding phase in this study, durvalumab 30 mg/kg and tremelimumab 1 mg/kg every 4 weeks was selected across all weight ranges as the RP2D for evaluation in the dose-expansion phase. Target exposure of durvalumab appeared to have been achieved for all patients with an evaluable AUC in the dose-expansion phase and was similar to that of the D30 + T1 (≥35 kg) dose-finding cohort. Achievement of the target tremelimumab exposure for patients with a measurable AUC (_0–28 and 0-14_) occurred for all patients. PK parameters were equivalent in both disease cohorts. The mean concentration-time profiles for the patients with sarcoma or a solid tumor showed no appreciable difference in PK results for these two disease cohorts. Geometric mean serum concentrations for durvalumab and tremelimumab in the dose-expansion phase are shown in **Supplementary Figure 4**; the complete summary of PK parameters for durvalumab and tremelimumab in the dose-expansion phase are summarized in **Supplementary Tables 6**, **7**, respectively.

### 3.3 Safety

In the D20+T1 cohort of the dose-finding phase, 9 of 10 (90.0%) patients experienced an AE of any grade, 4 (40.0%) had an AE of grade 3 or 4, and 5 (50.0%) had an AE considered possibly related to either study treatment by the investigator (**Supplementary Table 8**). The most common treatment-related AEs reported at D20+T1 are listed in **Table 2**. In the D30+T1 cohort, 18 of 19 (94.7%) patients experienced an AE of any grade, 6 (31.6%) had an AE of grade 3 or 4, and 12 (63.2%) had an AE considered by the investigator as possibly related to either study treatment (**Supplementary Table 8**). The most common treatment-related AEs reported at D30+T1 are listed in **Table 2**. Across the dose-finding phase, six grade 3 or 4 AEs possibly related to treatment were reported and included the following: dehydration, transverse sinus thrombosis, nausea, elevated aspartate aminotransferase, elevated amylase, and elevated lipase. A single patient in the D30+T1 cohort experienced grade 2 diarrhea and colitis (both conditions characterized as serious). This patient was treated with a high-dose corticosteroid regimen, with resolution of all toxicities. Consequently, tremelimumab was suspended after three cycles of combination treatment at D30+T1, but durvalumab was successfully resumed for this patient after resolution of this event.

**Table 2:** Treatment-related adverse events in the dose-finding phase.*

Adverse event, n (%)	D 20 mg/kg + T 1 mg/kg (n = 10)		D 30 mg/kg + T 1 mg/kg (n = 19)	
	Related to D only	Related to D and T	Related to D only	Related to D and T
Abdominal pain	0	0	0	1 (5.3)
Alanine aminotransferase increased	0	0	1 (5.3)	3 (15.8)
Amylase increased	0	0	0	1 (5.3)
Anemia	1 (10.0)	0	1 (5.3)	0
Aspartate aminotransferase increased	0	0	0	2 (10.5)
Blood alkaline phosphatase increased	0	0	0	1 (5.3)
Bronchospasm	1 (10.0)	0	0	0
Colitis	0	0	0	1 (5.3)
Constipation	0	0	1 (5.3)	0
Contusion	0	0	0	1 (5.3)
Cough	0	0	0	1 (5.3)
Decreased appetite	0	0	0	1 (5.3)
Dehydration	0	0	0	1 (5.3)
Dermatitis atopic	0	0	1 (5.3)	0
Diarrhea	0	0	2 (10.5)	1 (5.3)
Dyspnea	0	0	0	1 (5.3)
Fatigue	1 (10.0)	0	1 (5.3)	0
Gastroesophageal reflux disease	1 (10.0)	0	0	1 (5.3)
Headache	0	0	2 (10.5)	0
Hot flush	0	0	1 (5.3)	0
Hyperglycemia	1 (10.0)	1 (10.0)	1 (5.3)	0
Hypomagnesemia	0	0	1 (5.3)	0
Hypophosphatemia	0	1 (10.0)	0	0
Hypothyroidism	0	0	3 (15.8)	1 (5.3)
Leukopenia	0	0	2 (10.5)	0
Lipase increased	0	0	0	1 (5.3)
Myalgia	0	0	2 (10.5)	0
Nausea	0	0	2 (10.5)	1 (5.3)
Neutropenia	0	1 (10.0)	0	0
Neutrophil count decreased	0	0	1 (5.3)	1 (5.3)
Oral pain	1 (10.0)	0	0	0
Osteitis	1 (10.0)	0	0	0
Platelet count decreased	0	0	0	1 (5.3)
Pruritus	0	0	1 (5.3)	0
Pyrexia	0	0	0	1 (5.3)
Rash	1 (10.0)	0	0	0
Rash maculopapular	0	0	1 (5.3)	0
Skin depigmentation	0	0	1 (5.3)	0
Thrombocytopenia	0	0	1 (5.3)	0
Transverse sinus thrombosis	0	0	0	1 (5.3)
Vitiligo	0	0	1 (5.3)	0
Vomiting	1 (10.0)	1 (10.0)	0	0
White blood cell count decreased	0	0	1 (5.3)	0

In the dose-expansion phase, 19 of 21 (90.5%) patients experienced an AE of any grade, 10 (47.6%) had an AE of grade 3 or 4, and 16 (76.2%) had an AE considered by the investigator as possibly related to study treatment (**Supplementary Table 8**). The most common treatment-related AEs reported in the dose-expansion phase are listed in **Table 3**. Within the dose-expansion phase, three grade 3 or 4 AEs possibly related to treatment were recorded and included anemia, anorexia, and ascites; a single grade 3 AE of thrombocytopenia, possibly related to durvalumab, was also reported. Three patients experienced an immune-mediated AE, of which one was treatment related. One patient experienced grade 3 pneumonitis that was treated with a high-dose corticosteroid regimen and reported as resolved. A second patient experienced grade 2 hypothyroidism, treated with supplemental hormone replacement therapy (event ongoing at the time of study closure), and a third patient experienced grade 2 myositis, treated with a high-dose corticosteroid regimen (event ongoing at the time of study closure). Across both treatment phases, the incidence of grade 3 or 4 AEs possibly related to treatment was 35%. Overall, most events were low grade (grade 1–2) and manageable with no grade 5 events.

**Table 3:** Treatment-related adverse events in the dose-expansion phase.*

Adverse event, n (%)	Sarcoma (n = 11)		Solid tumors (n = 10)	
	Related to D only	Related to D and T	Related to D only	Related to D and T
Abdominal pain	0	2 (18.2)	1 (10.0)	1 (10.0)
Alanine aminotransferase increased	0	1 (9.1)	0	0
Anemia	0	2 (18.2)	0	3 (30.0)
Ascites	0	1 (9.1)	0	0
Aspartate aminotransferase increased	0	0	0	1 (10.0)
Asthenia	0	0	0	2 (20.0)
Blood lactate dehydrogenase increased	0	0	0	1 (10.0)
Decreased appetite	0	0	0	3 (30.0)
Diarrhea	0	0	1 (10.0)	1 (10.0)
Erythema	0	0	0	1 (10.0)
Fatigue	0	0	0	1 (10.0)
Gamma-glutamyltransferase increased	0	0	0	1 (10.0)
Headache	0	1 (9.1)	0	1 (10.0)
Hemolysis	0	1 (9.1)	0	0
Hemolytic anemia	0	1 (9.1)	0	0
Hypermagnesemia	0	0	0	1 (10.0)
Hyperphosphatemia	0	0	0	1 (10.0)
Hypoalbuminemia	0	0	0	1 (10.0)
Hypomagnesemia	0	0	0	1 (10.0)
Hypophosphatemia	0	0	0	1 (10.0)
Hypothyroidism	0	1 (9.1)	0	1 (10.0)
Insomnia	1 (9.1)	0	0	0
Lymphocyte count decreased	0	1 (9.1)	0	0
Nausea	0	0	1 (10.0)	0
Neutropenia	0	1 (9.1)	0	0
Platelet count decreased	0	0	1 (10.0)	0
Pneumonia	0	0	1 (10.0)	0
Pruritus	0	1 (9.1)	0	0
Pyrexia	2 (18.2)	3 (27.3)	0	1 (10.0)
Rash	0	0	1 (10.0)	1 (10.0)
Stomatitis	0	1 (9.1)	0	0
Thrombocytopenia	0	1 (9.1)	0	1 (10.0)
Weight increased	0	0	1 (10.0)	0
White blood cell count decreased	0	1 (9.1)	0	0

### 3.4 Antitumor activity and survival endpoints

Two patients in the dose-finding phase, one with osteosarcoma (treated at D20+T1) and one with renal cell carcinoma (treated at D30+T1), achieved a partial response with a duration of >1 year. The patient with osteosarcoma had received 31 cycles of treatment, with subsequent discontinuation of durvalumab maintenance therapy due to thoracic resection of a residual mass. At the time of study closure, the patient remained disease free and has not received any additional treatment. The patient with renal cell carcinoma received 53 cycles of treatment and was transitioned to the post-treatment access program; this patient remains in a partial response at the time of study closure. Stable disease was reported for two patients with osteosarcoma and chordoma, with disease control lasting 3 and 9 months, respectively.

**Figure 1:**
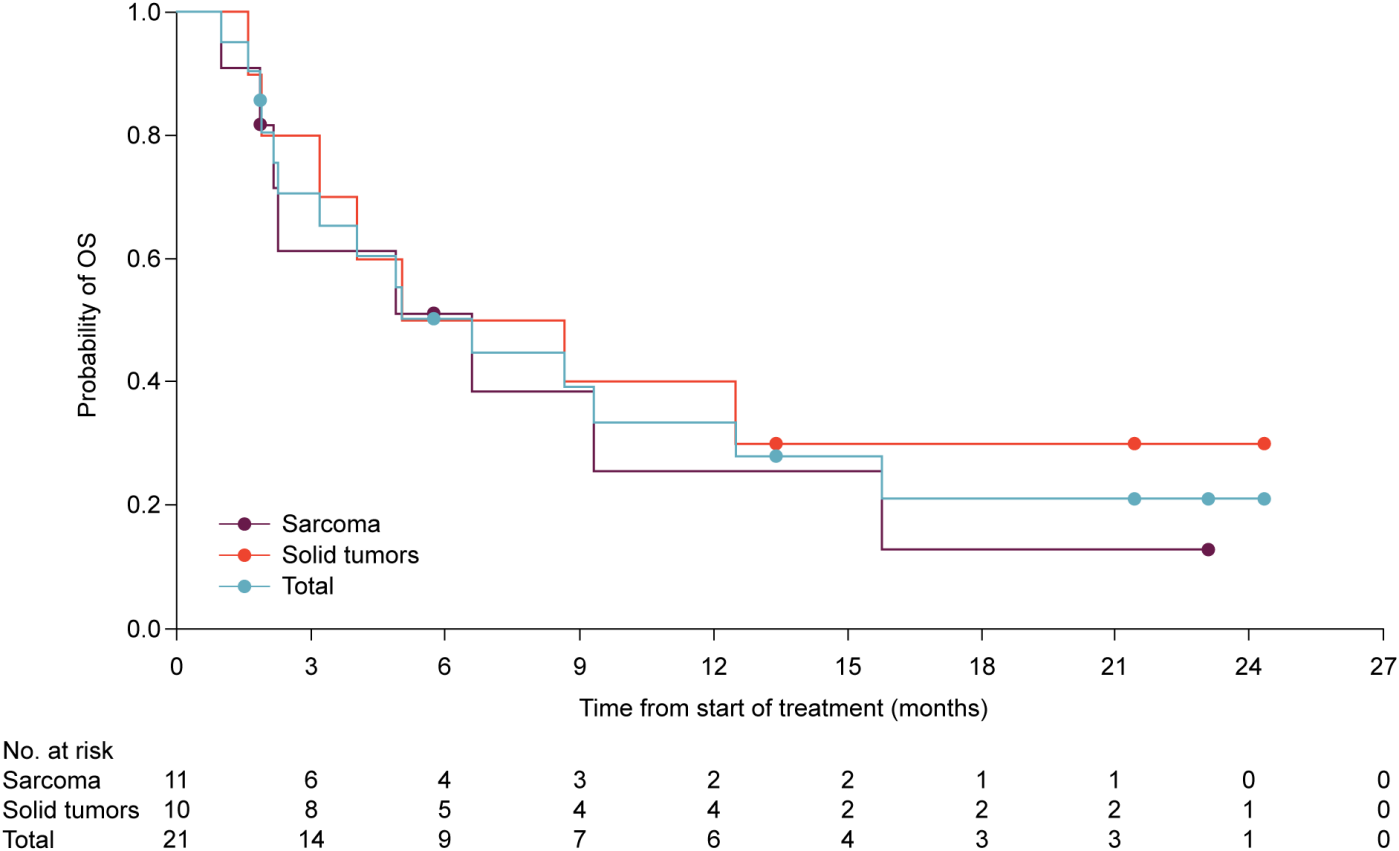
Kaplan-Meier plot of overall survival for patients in the dose-expansion phase. Median OS (90% confidence interval) was 6.6 months (1.9–15.8) in the sarcoma cohort (8 events among 11 patients), 6.9 months (1.6–not reached) in the solid tumors cohort (7 events among 10 patients), and 6.6 months (2.3–12.5) for all patients (15 events among 21 patients). Circles indicate censored observations.

In the dose-expansion phase, one patient achieved an objective response, yielding an ORR of 5.0% among 20 patients evaluable for response (**Table 4**). No patient in the sarcoma cohort achieved an objective response, and the cohort was not expanded further. The median PFS for the sarcoma cohort was 1.7 months (90% CI, 1.6–1.9) with a 12-month PFS rate of 9.1%. The median OS was 6.6 months (90% CI, 1.9–15.8), with a 12-month survival rate of 25.6% (**Figure 1**). In the solid tumor cohort (n = 10), one patient with chordoma had a confirmed partial response lasting 10.8 months. The median PFS for the solid tumor cohort was 1.7 months (90% CI, 0.9–2.8) with a 12-month PFS rate of 10.0%. The median OS was 6.9 months (90% CI, 1.6–not reached) with 12- and 24-month survival rates of 40.0% and 30.0%, respectively. Two patients achieved stable disease, with disease control at 24 weeks with durations reported as 19 months (undifferentiated sarcoma) and 7 months (renal cell carcinoma).

**Table 4:** Best objective response in the dose-expansion phase (evaluable patients).

Response status	Best objective response, n (%)	Sarcoma (n = 11)	Solid tumors (n = 9)	Total (N = 20 evaluable patients)
Response	Complete response (CR)	0	0	0
	Partial response (PR)	0	1 (11.1)	1 (5.0)
Non-response	Unconfirmed CR or PR	0	0	0
	Stable disease ≥7 weeks	1 (9.1)	1 (11.1)	2 (10.0)
	Disease progression	10 (0.9)	7 (77.8)	17 (85.0)
	RECIST progression	6 (54.5)	6 (66.7)	12 (60.0)
	Death*	4 (36.4)	1 (11.1)	5 (25.0)

*One patient in the sarcoma cohort died before having an initial disease assessment.

RECIST, Response Evaluation Criteria in Solid Tumors.

### 3.5 Immunogenicity and immune response data

Across both study phases, 17 patients were evaluable for ADA response to durvalumab and 10 for response to tremelimumab; evaluable patients included those who had received at least one dose of study treatment and had a measurement at baseline and a follow-up measurement post baseline. Three patients had a single ADA response reported at baseline (n = 2, durvalumab; n = 1, tremelimumab) and were ADA negative for the remainder of the study. No ADA responses were reported for the remaining ADA-evaluable patients.

Markers of immune cell activation during the dose-finding and dose-expansion phases were analyzed for 41 patients. Of these, 24 patients (12 in each phase) had evaluable data defined as a timepoint at baseline and at day 8 following treatment with the combination of durvalumab and tremelimumab. A transient increase in the percentage of CD4+Ki67+ T cells was observed in 100% of the evaluable patients (**Supplementary Figure 3**) at day 8 following durvalumab plus tremelimumab for both the dose-finding phase (cycle 2, day 8) and the dose-expansion phase (cycle 1, day 8) that returned to baseline 4 weeks after dosing (**Table 5**; **Supplementary Figure 5**). This immune cell activation pattern at day 8 following an initial cycle of the combination regimen is similar to that observed in comparable cohorts in a phase Ib study of adult patients with NSCLC, specifically the cohorts (as aggregated data) of patients who received durvalumab at 10, 15, and 20 mg/kg in combination with tremelimumab 1 mg/kg (15). Although not conducted as formal comparative analysis, the median percentage increase in CD4+Ki67+ T cells reported following an initial cycle of the combination in adults at day 8 was reported as 275%, whereas the magnitude of the median rise observed in pediatric patients receiving the R2PD regimen in the dose-expansion phase, at the same timepoint, was 113% (**Table 5**). A similar result, however, was not consistently observed with a rise in CD8+Ki67+ T cells at day 8 within the pediatric cohorts (**Supplementary Figure 5**). In contrast, a statistically significant rise in CD8+Ki67+ T cells was observed in the NSCLC cohorts at 1 week following administration of the combination. The median percentage increase in CD8+Ki67+ T cells at day 8 was 98% in adult patients with NSCLC, whereas the median increase for expansion reported for the three pediatric cohorts was –40%, 20% and 66.7% (**Table 5**; **Supplementary Figure 6**). Flow cytometry data were available for five of the seven patients who experienced disease control, including two with a partial response and three experiencing stable disease. There was no clear distinction in the immune cell activation profiles between the patients who achieved a partial response or stable disease and the aggregated data for patients experiencing progressive disease (**Supplementary Figure 7**).

**Table 5:** Summary of percentage changes in T cell absolute count (cells/mm^3^) from baseline in pediatric and adult cohorts.

Median % change from baseline (day 8) (25^th^, 75^th^ percentiles)	D 20 mg/kg + T 1 mg/kg (C2) Dose-finding phase	D 30 mg/kg + T 1 mg/kg (C2) Dose-finding phase	D 30 mg/kg + T 1 mg/kg (C1) Dose-expansion phase	D 10 mg/kg, D 15 mg/kg, and D 20 mg/kg + T 1 mg/kg Adult NSCLC cohort^‡^
CD4+Ki67+ T cell absolute count*	153.1 (58.0, 925.0) n = 6	118.2 (65.1, 215.0) n = 5	112.8 (69.2, 352.5) n =12	275 (140.6, 400)
CD8+Ki67+ T cell absolute count*	66.7 (–35.2, 900.0) n = 5	–40.0 (–57.0, 318.3) n = 5	20.0 (–25.0, 88.9) n = 7	98.8 (23.3, 237.5)
CD4+ T cell absolute count†	12.8 (–6.5, 63.8) n = 6	16.1 (–13.2, 52.7) n = 6	–12.5 (–20.5, 2.9) n = 12	2.2 (–12.5, 23.4)
CD8+ T cell absolute count†	30.1 (–14.6, 60.9) n = 6	13.8 (–18.0, 71.2) n = 6	–23.3 (–37.8, –10.3) n = 12	–10.7 (–26.1, 10.6)

*For the Ki67+ analysis, samples were collected from 17 patients in the dose-finding phase and 14 patients in the dose-expansion phase; values in the table reflect the number of patients (n) with a paired set of analyzed samples collected on days 1 and 8 (following the administration of D+T).

†For the TBNK analysis, samples were collected from 19 patients in the dose-finding phase and 14 patients in the dose-expansion phase; values in the table reflect the number of patients (n) with a paired set of analyzed samples collected on days 1 and 8 (following the administration of D+T).

‡A total of 145 samples were collected from adult patients with NSCLC in the dose-escalation and dose-expansion phase; details on the number of patients with a paired set of analyzed samples collected on days 1 and 8 (following the administration of D+T) are not available.

C, cohort; D, durvalumab; T, tremelimumab.

### 3.6 Biomarker analyses

**Figure 2:**
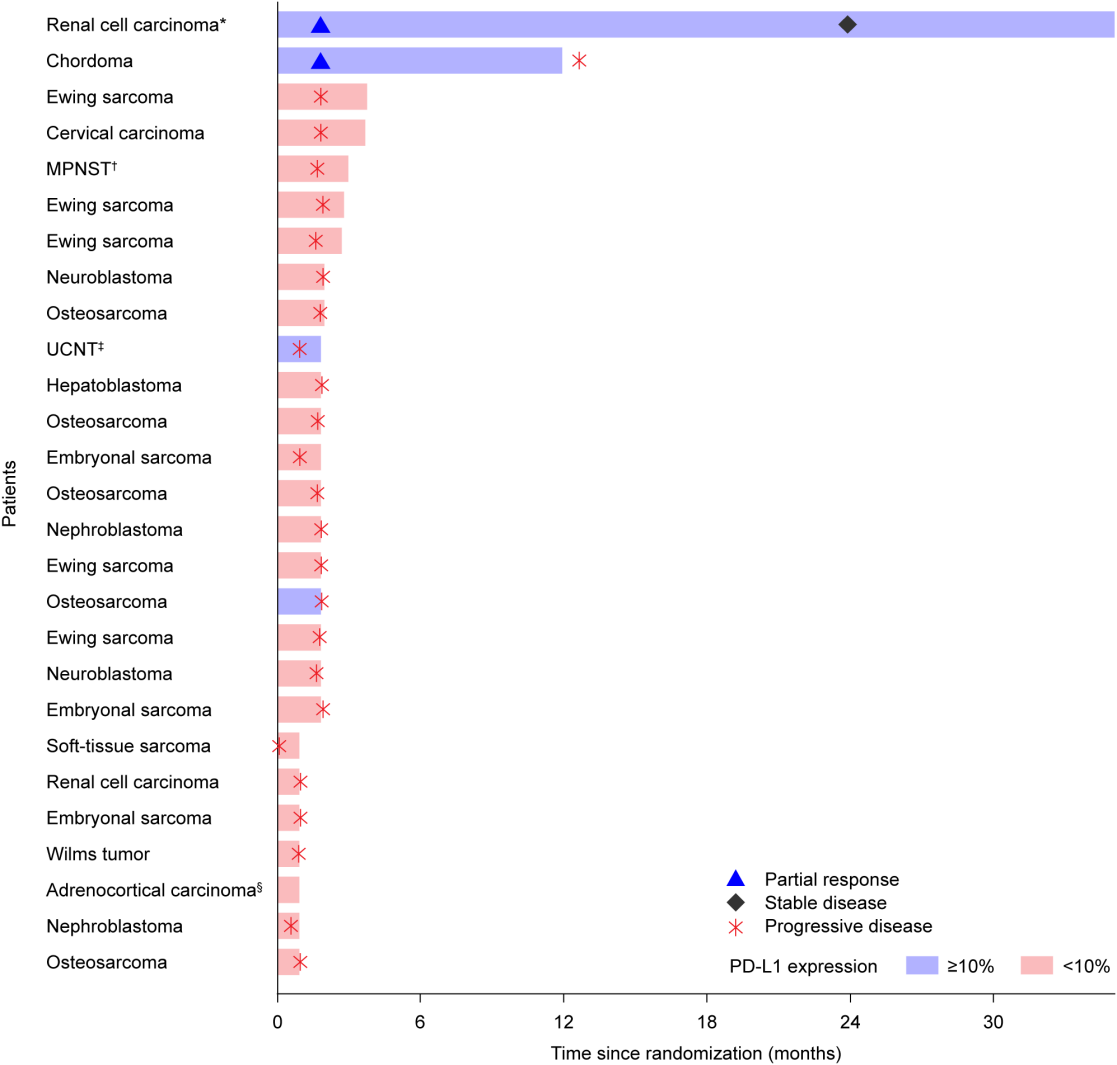
Swimmer plot of best overall response by tumor PD-L1 expression and tumor type. PD-L1 expression was assessed by immunohistochemistry in pretreatment tumor tissue samples using the investigational VENTANA PD-L1 (SP263) Assay (Roche Diagnostics, Tucson, AZ, USA). PD-L1 expression was scored using a TAP score (16), which was determined by visually aggregating/estimating the area covered by PD-L1–positive tumor cells and tumor-associated immune cells relative to the total tumor area, using a cutoff of 10%. *For the patient with renal cell carcinoma, although the last tumor assessment was recorded as stable disease, the investigator confirmed that the patient was still in response (partial response) at the time of study closure. §Not evaluable. PD-L1, programmed death ligand 1.

As an exploratory analysis, best overall response by baseline PD-L1 expression and tumor type was assessed. A total of 27 patients had tumor samples evaluable for PD-L1 expression at baseline, of whom four (14.8%) had tumor PD-L1 expression ≥10% reflected by renal cell carcinoma, chordoma, undifferentiated carcinoma of the nasopharyngeal tract, and osteosarcoma (**Figure 2**). In this analysis, two neuroblastoma samples demonstrated PD-L1 expression, both as ≤10%, and within the 16 sarcoma samples tested (including malignant peripheral nerve sheath tumor and chordoma), eight were confirmed as PD-L1+ but with only two recorded as ≥10%. Two of the three patients (renal cell carcinoma, chordoma) with a confirmed partial response had a tumor PD-L1 expression ≥10%.

## 4 Discussion

This is the first phase I/II study to evaluate the combination of durvalumab and tremelimumab in pediatric patients with solid tumors. The study population was heavily pretreated and had disease characteristics at study entry representative of pediatric patients with advanced solid tumors. In the dose-finding phase, based on safety data and PK analyses, the RP2D of durvalumab 30 mg/kg and tremelimumab 1 mg/kg provided an adult-equivalent drug exposure for pediatric patients. At the RP2D, the safety profile was consistent with the known safety profile of durvalumab in combination with tremelimumab in adult patients (13, 14), and no new safety signals were identified in our pediatric population. In the prior ADVL1412 study, 98% of study patients receiving the nivolumab/ipilimumab regimen experienced at least one treatment-related AE, with 38% of grade 3–4 AEs possibly attributed to treatment for patients receiving the RP2D (8). The safety profile observed for patients receiving the durvalumab/tremelimumab regimen appears comparable to that for the previous dual ICI regimen evaluated in children, with a similar overall incidence and pattern of AEs reported in both studies during the study treatment period; in this study, the incidence of grade 3–4 AEs possibly attributed to study treatment across both treatment phases was 35%.

To date, limited antitumor activity with ICI monotherapies and dual PD-(L)1 and CTLA-4 inhibitor regimens has been observed in pediatric solid tumors (3, 6–8, 10–12). Across four prior studies evaluating ICI monotherapy regimens in children with solid tumors and lymphoma, the collective ORR was 3.7% for the solid tumor population (nine responses in 244 patients) (17). In the expanded phase of the ADVL1412 study (8), the combination of nivolumab and ipilimumab was evaluated initially in patients with relapsed/refractory solid tumors followed by evaluation of the RP2D (nivolumab 3 mg/kg and ipilimumab 1 mg/kg, both administered every 4 weeks) in patients restricted to a sarcoma cohort. The ORR with the R2PD regimen was reported as 3.6% with two durable partial responses lasting at least 36 months in the sarcoma cohort; no responses were reported in the solid tumor phase. Stable disease was reported for four patients receiving the combination regimen (two patients with rhabdomyosarcoma and one patient each with nasopharyngeal carcinoma and myofibroblastic tumor) for a median duration of 5.6 months. Acknowledging the limitation of a cross-trial comparison, the activity of the two ICI (PD-[L]1/CTLA-4 inhibitor) combination regimens appears comparable, with similar patterns of disease control (partial response, stable disease) reported for both ADVL1412 and this study. The ICI combination of nivolumab (3 mg/kg) and ipilimumab (1 mg/kg) was also evaluated in patients with high-grade central nervous system tumors (ages 0.5–21 years) in the CheckMate 908 study, which reported no improvement in PFS and OS outcomes for patients with newly diagnosed diffuse intrinsic pontine glioma and relapsed/refractory central nervous system malignancies compared with historical survival data (9). The two partial responses reported in the ADVL1412 study occurred in a 25-year-old patient (alveolar rhabdomyosarcoma) and an 18-year-old patient (Ewing sarcoma). In this study, all three responses were reported in children aged <18 years, specifically, at ages 17 (osteosarcoma), 15 (chordoma), and 11 (renal cell carcinoma). Unfortunately, to date, no objective responses have been reported with any ICI combination regimen in young children (age <10 years) with a solid tumor (8, 17).

In the dose-finding and dose-expansion phases, data from all evaluable pediatric patients showed elevated levels of CD4+Ki67+ (proliferating) T cells at day 8 following initial combined durvalumab and tremelimumab administration, consistent with the immune activation profile observed in adult patients with NSCLC and suggesting similar target engagement and downstream pharmacodynamic effects in both populations (15). Similar increases could not be identified consistently for the Ki67+CD8+ T cell population in the pediatric cohorts but were more consistently observed in selected adult NSCLC cohorts. Although this was the first study conducted in a pediatric population to characterize immune responses with the administration of a combination ICI regimen, a clear conclusion about differences between these two populations is limited by the overlapping CIs and small sample size of the available data.

A robust expansion of proliferating CD8+ T cells is a critical response to immune-checkpoint inhibition leading to an enhanced adaptive immune response, specifically due to the activation of exhausted T cells (18). The effectiveness of the CD8+ response is highly dependent on conditions within the tumor microenvironment and the presence of adverse conditions such as abnormal vasculature, high interstitial pressure, and hypoxia—which are generally considered unfavorable for CD8+ cells, may explain the lack of a clinical response to an ICI regimen. Pediatric tumors, other than lymphoma (Hodgkin lymphoma and non-Hodgkin lymphoma), are generally classified as “cold tumors”; the presence of a sparse CD8+ T-cell tumor infiltration is considered the predominant phenomenon observed in these tumor types. 

For pediatric studies that include biomarker testing as part of the evaluation of a PD-(L)1 inhibitor, the reported PD-L1 positive expression rates range from 14% to 35%, with the highest expression rates reported in study cohorts of lymphoid malignancies (Hodgkin lymphoma and non-Hodgkin lymphoma) (6, 8, 10). Results from an analysis of 451 archived pediatric tumor samples (collected from two institutions) demonstrated the highest PD-L1 expression rates in Burkitt’s lymphoma (80%), glioblastoma multiforme (35%), and neuroblastoma (14%); however, low expression levels (≤1%) were reported in this analysis for sarcoma samples (19). In this study, PD-L1 expression using a ≥10% cutoff was reported in approximately 15% of 27 evaluable tumors (renal cell carcinoma, chordoma, undifferentiated carcinoma of the nasopharyngeal tract, and osteosarcoma). To date, data from pediatric ICI studies that have included PD-L1 biomarker testing have not established a clear correlation between PD-L1 expression and response to ICI treatment; however, given the overall low rates of PD-L1 expression and low rates of clinical responses reported, there are inherent limitations in establishing any conclusion regarding the predictive value of PD-L1 as a biomarker in the pediatric population.

Results from genetic testing were available for the adolescent patient with osteosarcoma who experienced a durable partial response; interestingly, this patient was found to have a germline *TP53* mutation. Osteosarcoma occurs more often in patients with Li-Fraumeni syndrome, and a high somatic *TP53* mutation rate (9.5%) has been reported in patients aged <30 years with osteosarcoma (20). While previous pediatric studies of ICIs have demonstrated limited activity in osteosarcoma, there have been other single cases of durable partial responses reported in adult trials (21, 22). However, no specific disease characteristics or biomarker were identified to correlate with these responses. There are also reports of durable responses to ICI therapy reported in adult patients with various solid tumors and confirmed Li-Fraumeni syndrome (23). Genetic testing was also performed for the patient with renal cell carcinoma, characterized as a long-term responder; however, no clinically relevant mutations were reported by the investigator for this patient.

In this study, all three objective responses were reported in children <18 years of age, specifically at ages 17 (osteosarcoma), 15 (chordoma), and 11 (renal cell carcinoma). Two of these responding patients (renal cell carcinoma [RCC] and chordoma) had tumor PD-L1 expression >10%; the sample from the third patient unfortunately was inadequate to characterize PD-L1 expression. Additional clinical benefit is reflected for these specific tumor types as evident by stable disease reported in a second patient with renal cell carcinoma (11 years) lasting 7 months and in a second patient (7 years) with chordoma lasting 10 months. Interestingly, these are also tumor types for which there is response data in adults with relapsed chordoma and relapsed renal cell carcinoma who have received a PD-L1/CTLA-4 regimen. Specifically, in a study of adult patients (18 years or older) with relapsed sarcoma receiving the same durvalumab and tremelimumab regimen used in this study, a single partial response was reported and three patients experienced stable disease within the chordoma cohort (n= 5) using immune-related Response Criteria (irRC); however, no objective responses were reported within the osteosarcoma cohort (24). Responses have been reported in adult patients with relapsed renal cell carcinoma in the TITAN-RCC study who initially received nivolumab monotherapy, followed by boost dosing consisting of two or four doses of ipilimumab with a disease control rate of 52% of patients within the group of relapsed patients who initially had progressive disease with nivolumab monotherapy, characterized by complete response, partial response and stable disease rates of 7%, 17%, and 29% of patients, respectively; median duration of response was 18.8 months and 7.1 months for patients with stable disease (25). These findings suggest the possibility of similar biological features contributing to responses observed for the same tumor in the adult and pediatric setting, possibly due to the relevance of the PD-L1 biomarker expressed in both of these tumor types and the potential for similar immunological responses with the targeting of the PD-L1/CTLA-4 pathways. The durability of the partial response for the patient with RCC as well as the partial response observed in our patient with osteosarcoma, however, suggests another possible mediator or mechanism independent of an immune checkpoint that may contribute to the differing response reported in the adult setting for relapsed tumors that have been treated with a dual immune checkpoint inhibitor regimen.

Advances have been made in pediatric oncology in the last few years leading to the approval of ICI regimens in select childhood cancers, based on limited data from either studies evaluating an ICI for a specific solid tumor that included pediatric, including adolescent young adult, patients (i.e., alveolar soft part sarcoma), or through extrapolation for children (≥12 years) from studies conducted in adults with solid tumors who have been identified as having a specific disease characteristic (i.e., microsatellite instability–high/deficient mismatch repair and high tumor mutation burden) (26) or with a potential presentation in a child (i.e., Merkel cell carcinoma) (27–30). Additionally, ICI monotherapy regimens are approved for lymphoid malignancies in children, specifically as treatment for refractory classic Hodgkin lymphoma and primary mediastinal B-cell lymphoma in children (27)—two tumor types that have demonstrated responses to various PD-L1 inhibitors due the presence of the 9p24.1 amplification resulting in increased expression of the PD-1 ligand (31). Unfortunately, for pediatric patients with a solid tumor, the use of approved ICI regimens is limited to a small number of patients with rare solid tumors, including those malignancies containing specific tumor characteristics that are not detected in most childhood cancers.

In summary, our study showed that the adult-equivalent RP2D of durvalumab combination with tremelimumab had a tolerable and manageable safety profile in pediatric patients with relapsed/refractory solid tumors; the safety profile was consistent with that reported in adult patients treated with the combination. The conclusion from our study is consistent with those of other studies showing limited antitumor activity in children with relapsed/refractory solid tumors (including sarcomas), when ICIs are given either as a monotherapy regimen or administered as a dual PD-(L)1 and CTLA-4 inhibitor regimen. Despite a lack of an efficacy signal indicating any responsiveness for a particular tumor type in this study, two highly durable partial responses were reported, with both patients remaining alive at the completion of the study. Childhood cancers have fewer mutations and are generally less inflammatory than adult tumors, meaning immune cells are not activated against pediatric tumors. Strategies to break the immune tolerance observed in pediatric tumors and to activate the host immune system prior to ICI challenge are required to extend activity beyond a small subset of patients.

## Data Availability

The original contributions presented in the study are included in the article/**Supplementary Material**. Further inquiries can be directed to the corresponding author.
