# Correction: Phase I/II, open-label, multicenter study of durvalumab in combination with tremelimumab in pediatric patients with advanced solid tumors

**DOI:** 10.3389/fonc.2026.1886923

**Published:** 2026-06-09

**Authors:** Darren Hargrave, Lynley V. Marshall, Nicolas André, Julie Krystal, Brian H. Ladle, Karen A. Robbins, Stephan Hois, Jon Armstrong, Sarah Donegan

**Affiliations:** 1 University College London Great Ormond Street Institute of Child Health and Great Ormond Street Hospital for Children, London, United Kingdom; 2 Pediatric and Adolescent Oncology Drug Development Unit, Oak Centre for Children and Young People, The Royal Marsden Hospital and Division of Clinical Studies, The Institute of Cancer Research, London, United Kingdom; 3 Pediatric Hematology and Oncology Department, Hôpital pour Enfant de La Timone, Assistance Publique - Hôpitaux de Marseille (AP-HM), and Reverse Molecular Pharmacology in Pediatric Oncology, Centre de Recherche en Cancérologie de Marseille (CRCM), Aix-Marseille Université, CNRS, INSERM, Marseille, France; 4 Zucker Hofstra School of Medicine, Department of Pediatrics, Cohen Children’s Medical Center, New Hyde Park, NY, United States; 5 Sidney Kimmel Comprehensive Cancer Center, Johns Hopkins University School of Medicine, Baltimore, MD, United States; 6 Global Patient Safety, Oncology, AstraZeneca, Gaithersburg, MD, United States; 7 Late-stage Development Oncology R&D, AstraZeneca, Gaithersburg, MD, United States; 8 Oncology Biometrics, AstraZeneca, Cambridge, United Kingdom; 9 Oncology R&D, AstraZeneca, Gaithersburg, MD, United States

**Keywords:** antitumor activity, biomarker analysis, combination therapy, cytotoxic T-lymphocyte associated protein 4 inhibitor, pharmacokinetics, programmed death ligand 1 inhibitor, solid tumors

There was a mistake in **Table 1** as published. The numerical values in the columns under the subheader Tumor types are misaligned; additionally, the footnote corresponding to the histology missing term is missing from the bottom of the table. The corrected **Table 1** appears below

**Table 1:** Demographic and disease characteristics of the patients at baseline (at time of study enrollment).

Characteristic	Dose-finding phase (n = 29)	Dose-expansion phase (n = 21)	Total (N = 50)
Age, median (range), year	11.0 (3–17)	14.0 (1–17)	11.5 (1–17)
Age group, n (%), year			
≥1 to <6	4 (13.8)	2 (9.5)	6 (12.0)
6 to <12	11 (37.9)	8 (38.1)	19 (38.0)
12 to <18	14 (48.3)	11 (52.4)	25 (50.0)
Sex, n (%)			
Male	14 (48.3)	10 (47.6)	24 (48.0)
Female	15 (51.7)	11 (52.4)	26 (52.0)
Race, n (%)			
Black or African American	3 (10.3)	0	3 (6.0)
American Indian or Alaska Native	0	1 (4.8)	1 (2.0)
Asian	1 (3.4)	2 (9.5)	3 (6.0)
White	19 (65.5)	14 (66.7)	33 (66.0)
Other	6 (20.7)	4 (19.0)	10 (20.0)
Ethnicity, n (%)			
Hispanic or Latinx	5 (17.2)	2 (9.5)	7 (14.0)
Not Hispanic or Latinx	24 (82.8)	19 (90.5)	43 (86.0)
Weight group, n (%), kg			
<35	11 (37.9)	7 (33.3)	18 (36.0)
≥35	18 (62.1)	14 (66.7)	32 (64.0)
Prior systemic anticancer therapies, n (%)			
Cytotoxic chemotherapy	27 (93.1)	19 (90.5)	46 (92.0)
Immunotherapy	6 (20.7)	4 (19.0)	10 (20.0)
Biologic therapy	3 (10.3)	0	3 (6.0)
Experimental therapy	2 (6.9)	2 (9.5)	4 (8.0)
Radiopharmaceuticals	1 (3.4)	0	1 (2.0)
Other*	11 (41.4)	7 (33.3)	18 (36.0)
Number of prior anticancer therapies, n (%)			
0	0	0	0
1	6 (20.7)	5 (23.8)	11 (22.0)
2	4 (13.8)	4 (19.0)	8 (16.0)
3	4 (13.8)	6 (28.6)	10 (20.0)
>3	13 (44.8)	4 (19.0)	17 (34.0)
Unknown	2 (6.9)	2 (9.5)	4 (8.0)
Prior radiotherapy, n (%)	15 (51.7)	16 (76.2)	31 (62.0)
Prior surgery, n (%)	28 (96.6)	16 (76.2)	44 (88.0)
Lansky or Karnofsky performance score, n (%)			
100	12 (41.4)	11 (52.4)	23 (46.0)
90	9 (31.0)	5 (23.8)	14 (28.0)
80	3 (10.3)	3 (14.3)	6 (12.0)
70	4 (13.8)	1 (4.8)	5 (10.0)
60	1 (3.4)	1 (4.8)	2 (4.0)
Time from initial diagnosis, n (%), months			
≤24	15 (51.7)	11 (52.4)	26 (52.0)
>24	14 (48.3)	10 (47.6)	24 (48.0)
Disease classification, n (%)			
Metastatic	18 (62.1)	15 (71.4)	33 (66.0)
Locally advanced	1 (3.4)	2 (9.5)	3 (6.0)
Both	8 (27.6)	3 (14.3)	11 (22.0)
Missing or not applicable	2 (6.9)	1 (4.8)	3 (6.0)
Tumor types, n (%)			
SarcomaI Bone (Osteosarcoma and Ewing’s sarcoma) Soft-Tissue Sarcoma Alveolar soft-part sarcoma Clear-cell sarcoma Embroynal rhabdomyosarcoma ETRAN-TR Fibromyxoid sarcoma MPNST Poorly differentiated chordoma Undifferentiated sarcoma Histology not recorded *	18 (62.1) 9 6 1 1 0 1 1 1 1 0 3	11 (52.4) 5 6 1 0 2 0 0 1 0 1 0	29 (58.0) 14 (48.2) 12 (41.3) (3 missing histology)
Neuroblastoma	3 (10.3)	0	3 (6.0)
Solid tumors Adrenocortical carcinoma Chordoma (one poorly differentiated) Clear-cell adenocarcinoma/carcinoma Lymphoepithelial carcinoma Hepatocellular carcinoma Malignant rhabdoid tumor Nephroblastoma Renal cell carcinoma Ovarian cystoadenocarcinoma Uveal melanoma UCNT Wilms Tumor	8 (27.6) 1 1 0 0 1 1 1 2 0 0 1 0	10 (47.6) 0 1 2 1 0 1 0 1 1 1 0 2	18 (36.0)

* Includes use of tyrosine kinase and mechanistic target of rapamycin (mTOR) inhibitors, a retinoid, and an immunomodulating imide drug (IMiD).

* Reflects instances where tumor type was not properly captured in the database.

ETRAN, Embroynal tumor with multi-layered rosettes; MPNST, Malignant peripheral nerve sheath tumor; UCNT, Undifferentiated nasopharyngal cancer.

**Table:** 

Tumor types, n (%)			
Sarcoma Bone (Osteosarcoma and Ewing sarcoma) Soft-tissue sarcoma Alveolar soft-part sarcoma Clear-cell sarcoma Embroynal rhabdomyosarcoma ETRAN-TR Fibromyxoid sarcoma MPSNT Poorly differentiated chordoma Undifferentiated sarcoma Histology not recorded *	18(62.1) 9 6 1 1 0 1 1 1 1 0 3	11(52.4) 5 6 1 0 2 0 0 1 0 1 0	29 (58.0) 14 (48.2) 12 (41.3) (3 missing histology)

* Includes use of tyrosine kinase and mechanistic target of rapamycin (mTOR) inhibitors, a retinoid, and an immunomodulating imide drug (IMiD).

* Reflects instances where tumor type was not properly captured in the database.

ETRAN, Embroynal tumor with multi-layered rosettes; MPNST, Malignant peripheral nerve sheath tumor; UCNT, Undifferentiated nasopharyngal cancer.

The original version of this article has been updated.

