# Supplementary material for: Phase I/II, open-label, multicenter study of durvalumab in combination with tremelimumab in pediatric patients with advanced solid tumors
Source: Front Oncol. 2026 May 15;16:1680081. doi: 10.3389/fonc.2026.1680081 (PMC13218898; doi:10.3389/fonc.2026.1680081)
Supplement: Supplementary file 1 [file DataSheet1.docx]

**Supplementary Material**

**Table of Contents**

Study investigators........................................................................................................................ 2

Supplemental methods..................................................................................................................3

TABLE S1 Representativeness of the study participants.............................................................. 4

FIGURE S1 Patient disposition in the dose-finding phase ............................................................6

FIGURE S2 Patient disposition in the dose-expansion phase ......................................................8

Summary of PK Analyses ..............................................................................................................9

FIGURE S3 Geometric mean serum concentrations over time – dose-finding phase ................11

TABLE S4 Summary of PK parameters for durvalumab in the dose-finding phase ..................13

TABLE S5 Summary of PK parameters for tremelimumab in the dose-finding phase ............... 14

FIGURE S4 Geometric mean serum concentrations over time – dose-expansion phase ..........15

TABLE S6 Summary of PK parameters for durvalumab in the dose-expansion phase ............. 17

TABLE S7 Summary of PK parameters for tremelimumab in the dose-expansion phase ......... 18

TABLE S8 Adverse events assessed by the investigator............................................................ 19

FIGURE S5 Percentage change from baseline in CD4+Ki67+ T cell absolute counts in evaluable patients ....................................................................................................................... 20

FIGURE S6 Percentage change from baseline in CD8+Ki67+ T cell absolute counts in evaluable patients ....................................................................................................................... 21

FIGURE S7 Percentage change from baseline in CD4+Ki67+ T cells (A) and CD8+Ki67+ T cells (B) in responding and nonresponding patients........................................................................... 22

Study investigators

| **Investigator** | **Institution** |
| --- | --- |
| Amoroso, Loredana | Istituto di Ricovero e Cura a Carattere Scientifico Istituto Giannina Gaslini, Genova, Italy |
| André, Nicolas | Hôpital pour Enfant de La Timone, Assistance Publique - Hôpitaux de Marseille (AP-HM), Marseille, France |
| Collins, Natalie | Children's Hospital, Boston, Massachusetts, USA |
| Doz, François | Institut Curie and Paris Descartes University, Paris, France |
| Elliott, Martin | Leeds Teaching Hospitals, NHS Trust, Leeds, United Kingdom |
| Fischer, Matthias | Children's Hospital and Center for Molecular Medicine, Medical Faculty, University of Cologne, Cologne, Germany |
| Hargrave, Darren | Great Ormond Street Hospital for Children, London, UK |
| Krystal, Julie | Cohen Children's Medical Center, New Hyde Park, New York, USA |
| Ladle, Brian H. | Sidney Kimmel Comprehensive Cancer Center, Johns Hopkins University School of Medicine, Baltimore, Maryland, USA |
| Luksch, Roberto | Fondazione Istituto di Ricovero e Cura a Carattere Scientifico Istituto Nazionale dei Tumori, Milan, Italy |
| Marshall, Lynley V. | Pediatric and Adolescent Oncology Drug Development Unit, Oak Centre for Children and Young People, The Royal Marsden Hospital and Division of Clinical Studies, The Institute of Cancer Research, London, UK |
| McNall, Rene | University of Oklahoma Health Sciences Center, Oklahoma City, Oklahoma, USA |
| Rubio-San-Simón, Alba | Niño Jesús Children's Hospital, Madrid, Spain |

Supplementary methods

**Study design modifications**

Modifications to the study design were made to reflect changes to the disease cohorts as approved by the Pediatric Committee of the European Medicines Agency. Key modifications to the study design were (i) removal of the cohorts with hematologic malignancies because of unfavorable results reported with the durvalumab plus tremelimumab combination in adult leukemia (acute myeloid leukemia/myelodysplastic syndrome), the emergence and preferential use of other regimens for patients with Hodgkin lymphoma (HL) and acute lymphoblastic leukemia, (ii) removal of the non-Hodgkin lymphoma (NHL) cohort because of feasibility challenges (no patients with NHL had been enrolled at the time of study completion), (iii) removal of the neuroblastoma cohort from the dose-expansion phase because of lack of antitumor activity in the dose-finding phase, and (iv) limiting the total enrollment in the solid tumor cohort in the dose-expansion phase to 10 patients because of feasibility challenges and lack of activity.

**Immune activation analyses**

Samples were analyzed on a BD FACSCanto^TM^ II flow cytometry system (BD Biosciences) using BD FACSDiva^TM^ software. Cell counts per µL of blood for T, B, and NK cells were established from the TBNK assay (R&D Systems, Minneapolis, MN, USA) using a lyse no wash protocol, and these counts were applied to the percentage values of corresponding gated populations in the proliferation and activation/memory panels to derive cells/µL blood values. Change from baseline for percentage and absolute count values were derived as the value on any nonbaseline study day minus the predose baseline result (i.e., day 1, prior to dosing) divided by the predose baseline result.

TABLE S1 Representativeness of the study participants.

| Cancer type | Solid tumors – sarcoma, neuroblastoma, and other solid tumors |
| --- | --- |
| Considerations related to: | |
| Sex | Data suggest that sex may be a biologic risk factor for childhood cancer, with a higher incidence in males than females for most pediatric cancers, including neuroblastoma, rhabdomyosarcoma, and osteosarcoma (1). Among 71,906 cases of childhood cancer in the Surveillance, Epidemiology, and End Results (SEER) database, 53% were male (1). |
| Age | The age at diagnosis varies depending on the tumor type, but most pediatric solid tumors are initially diagnosed between 1 and 5 years of age. Among children (aged 1–18 years) with bone sarcoma (osteosarcoma and Ewing sarcoma), the average age at presentation is 13.1 years (2). |
| Race/ethnicity | Racial and ethnic disparities exist among patients with pediatric solid tumors. Patients with Black race or Hispanic ethnicity are more likely to be diagnosed at later stages of disease, have differences in treatments, and have lower survival rates (3). Differences in incidence rates also vary by race depending on the tumor type. For example, Black children have slightly higher rates of osteosarcoma and rhabdomyosarcoma than White children, but have a slightly lower rate of neuroblastoma than White children (4). In the United States, among 47,337 patients with primary malignant sarcoma, 63.3% were White, 15.8% were Hispanic, 12.0% were Black, and 8.8% were American Indian-Alaskan Native and Asian Pacific Islander (5). |
| Geography | In 2020, among children 0–14 years of age, there were 206,362 newly reported cases of cancer worldwide, with 80,104 deaths (6). The global age-standardized incidence rate was 10.5, with the highest incidence rates in North America (17.3), Western Europe (16.9), and Australia and New Zealand (16.7) (6). However, the highest age-standardized mortality rates occurred in Eastern Africa (5.6), Central America (4.8), and Southeast Asia (4.5) (6). |
| Other considerations | Evaluating new treatments for cancer in children is more challenging than in adults because of the relative rarity of childhood cancer. Thus, feasibility challenges limit the ability to conduct large studies in pediatric patients with solid tumors. However, there are recognized inequities in childhood cancer research that need to be addressed in order to achieve adequate representation in clinical studies with respect to race and ethnicity among children with cancer (7). |
| Overall representativeness of the study | Our study enrolled slightly more female patients than male patients, in contrast to the higher incidence of male patients with pediatric solid tumors in a real-world population. However, pediatric solid tumors are commonly diagnosed in both sexes. The median age of the patients enrolled in our study was 11.5 years, with 50% in the range of 12 to <18 years. Taking into account that our study enrolled patients who had been previously treated, the age of our study population reflects that of a real-world pediatric population.  Our study was conducted in regions with the highest reported incidences of pediatric cancers (North America and Western Europe). However, due to the small size of this phase I/II study, the enrolled population did not represent a broad geographical region. Black and Asian patients, in particular, were under-represented in our study. |

**References**

1. Williams LA, Richardson M, Marcotte EL, Poynter JN, Spector LG. Sex-ratio among childhood cancers by single-year of age. *Pediatr Blood Cancer*. (2019) 66:e27620.
2. Kha ST, Sharma J, Kenney D, Daldrup-Link H, Steffner R. Assessment of the interval to diagnosis in pediatric bone sarcoma. *Pediatr Emerg Care*. (2023) 39:963–67.
3. Roberts B, Cooke-Barber J, Ingram MC, Danko M, Trudeau M, Glick RD, et al. Disparities in care of pediatric, adolescent, and young adult patients with solid tumors: a systematic review. *Pediatr Blood Cancer*. (2023) 70:e30355.
4. Marcotte E, Domingues A, Sample J, Richardson MR, Spector LG. Racial and ethnic disparities in pediatric cancer incidence among children and young adults in the United States by single year of age. Cancer. (2021) 127:3651–63.
5. Diessner BJ, Weigel BJ, Murugan P, Zhang L, Poynter JN, Spector LG. Associations of socioeconomic status, public vs private insurance, and race/ethnicity with metastatic sarcoma at diagnosis. *JAMA Netw Open*. (2020) 3:e2011087.
6. Huang J, Chan SC, Ngai CH, Lok V, Zhang L, Lucerno-Prisno DE 3rd, et al. Global incidence, mortality and temporal trends of cancer in children: a joinpoint regression analysis. *Cancer Med.* (2023) 12:1903–11.
7. Hunleth J, Burack S, Kaufman L, Mohrmann C, Shato T, Wiedenman E, et al. Inequities in childhood cancer research: a scoping review. *EJXC Paediatr Oncol*. (2024) 4:100171.

FIGURE S1 Patient disposition in the dose-finding phase.

33 patients screened*

4 excluded

- 3 screen failure
- 1 withdrew consent

29 patients received study treatment

19 patients at dose level 2 – D30 + T1

4 disease cohorts† and 2 weight bands

- 19 received durvalumab
- 12 received both durvalumab and tremelimumab

10 patients at dose level 1 – D20 + T1
4 disease cohorts† and 2 weight bands

- 10 received durvalumab
- 9 received both durvalumab and tremelimumab

19 patients terminated the study

19 patients discontinued treatment

- 17 disease progression
- 2 patient decision
  (including lost to follow- up)

10 patients terminated the study

10 patients discontinued treatment

- 8 disease progression
- 1 initiation of new anticancer treatment (surgical resection)
- 1 study completion‡

D20 + T1=durvalumab 20 mg/kg plus tremelimumab 1 mg/kg; D30 + T1=durvalumab 30 mg/kg plus tremelimumab 1 mg/kg. *Informed consent received. †Disease cohorts were neuroblastoma, sarcoma (bone), sarcoma (soft tissue), and solid tumors. ‡Patient transitioned to the post-treatment access program.

FIGURE S2 Patient disposition in the dose-expansion phase.

23 patients screened*

2 excluded

- 2 screen failure

21 patients received study treatment

10 patients with solid tumors – D30 + T1 (R2PD)

- 10 received durvalumab (30 mg/kg)
- 10 received both durvalumab (30 mg/kg) and tremelimumab (1 mg/kg)

11 patients with sarcoma – D30 + T1 (R2PD)

- 11 received durvalumab (30 mg/kg)
- 11 received both durvalumab (30 mg/kg) and tremelimumab (1 mg/kg)

10 patients discontinued treatment

- 10 disease progression

11 patients discontinued treatment

- 11 disease progression

10 patients terminated the study

11 patients terminated the study

D30 + T1=durvalumab 30 mg/kg plus tremelimumab 1 mg/kg. *Informed consent received.

Summary of PK analyses

Pharmacokinetic (PK) analyses included individual durvalumab and tremelimumab serum concentrations and PK parameters including maximum serum concentration (C_max_), minimum serum concentration (C_min_), area under the curve (AUC), and other parameters where appropriate. In the dose-finding phase, PK results are summarized by treatment and body weight (arm A: ≥35 kg; arm B: <35 kg). Geometric mean serum durvalumab concentrations for cycle 1 and tremelimumab concentrations from cycle 2 (first administration) are shown in Supplemental Figure 1, reflecting the two dose levels of durvalumab (20 mg/kg and 30 mg/kg) with a fixed dose of tremelimumab (1 mg/kg) evaluated in the dose-finding phase. Following the first dose, serum durvalumab concentrations peaked at the end of the 2-hour infusion period for all patients, declining thereafter through 28 days post dose. The levels were quantifiable for all patients evaluated with geometric mean C_min_ values as <50.0 μg/mL for the D20+T1 cohorts and >100 µg/mL for the D30+T1 cohorts following a single dose of durvalumab (Supplemental Table 4).

Geometric mean exposure was higher at 30 mg/kg compared with 20 mg/kg, with a trend toward higher mean concentrations in patients ≥35 kg than in patients <35 kg. For geometric mean AUC, C_max_, and C_min_, exposure in patients <35 kg was ~70% to 75% of the exposure for patients ≥35 kg (except where C_min_ was approximately half) for both the 20 and 30 mg/kg doses. Target durvalumab exposures of AUC_(0–28)_ ≥2105 day*µg/mL were achieved with the first dose for five of six patients in arm A who received D20+T1 and had reportable AUC_(0–28)_ (and for one additional patient for whom AUC_[0–28]_ was not calculable, but AUC_[0–14]_ was ≥2105 day*µg/mL). All three patients who received D20+T1 had reportable AUC_(0–28)_, and two of three values reached the exposure target. Patients treated with D30+T1 met or exceeded target systemic exposure in both arm A and arm B (in which AUC_[0–28]_ was evaluable); of 12 patients with calculable AUC_(0–28)_ (eight in arm A and four in arm B), all met systemic exposure targets.

Following the first dose of tremelimumab, serum concentrations peaked at the end of the 1-hour infusion period before declining through the last sample at 28 days post dose. At 28 days post dose, tremelimumab was quantifiable for all patients evaluated at geometric C_min_ values of ~3 to 4 µg/mL; C_min_ was not reportable for patients in arm B who received D20+T1 because there were no cycle 3 predose samples reported for this cohort (Supplemental Table 5). Tremelimumab concentrations were similar across weight groups. Mean tremelimumab levels appeared higher at the end of the infusion period for patients <35 kg treated with D20+T1 compared with other cohorts; notably, however, this was due to a single high value for one patient. Tremelimumab AUC, C_min_, and C_max_ were similar across the cohorts, and there were no clear trends related to body weight; geometric mean AUC_(0–28)_ values were within 5% of each other where comparisons could be made (30 mg/kg). Tremelimumab systemic exposures were achieved for all patients with calculable AUC_(0–28)_, including six patients in arm A and three patients in arm B. Overall, the PK results supported selection of the recommended phase II dose as 30 mg/kg durvalumab and 1 mg/kg tremelimumab for pediatric patients ≥35 kg and <35 kg, in fulfillment of specifications for adequate exposure as defined by protocol-specified criteria.

In the dose-expansion phase, PK results are summarized by tumor type (sarcoma vs. solid tumor); all patients received D30+T1 from the first cycle onward. Geometric mean serum durvalumab concentrations for cycle 1 of the dose-expansion phase and tremelimumab concentrations are shown in Supplemental Figure 2. Following the first dose, serum durvalumab peaked at the end of the infusion period for all patients, declining thereafter through 28 days post dose, where levels were quantifiable for all patients evaluated with geometric C_min_ as 108 and 78.3 µg/mL for patients with sarcoma and other solid tumors, respectively (Supplemental Table 6). Geometric mean concentration-time profiles were very similar for patients with sarcoma and other solid tumors, with no appreciable difference in PK results. Target durvalumab systemic exposures (i.e., AUC_[0–28]_ ≥2105 day*µg/mL) appeared to have been achieved for all patients with an evaluable AUC in the dose-expansion phase; AUC_(0–28)_ was reported for 14 patients, with all results >2105 day*µg/mL. Of seven patients with no reported AUC_(0–28)_, the AUC_(0–14)_ was >2105 day*µg/mL for five patients and was not calculable for two patients. Overall, durvalumab PK results in the dose-expansion phase were similar to those in the dose-finding phase, most closely matching the arm B D30+T1 cohort.

Following a dose of tremelimumab 1 mg/kg, serum concentrations peaked at the end of the 1-hour infusion period before declining through the last sample at 28 days post dose, where levels were quantifiable for all patients evaluated at a geometric C_min_ of 3.91 and 3.40 µg/mL for patients with sarcoma and other solid tumors, respectively (Supplemental Table 7). Target tremelimumab systemic exposures (i.e., AUC[0–28] ≥119.5 day*µg/mL) were achieved for all 15 patients with calculable AUC_(0–28)_. Of five patients with no reportable AUC_(0–28)_, two had no calculable AUC (<3 samples), and the remaining three had AUC_(0–14)_ ranging from 113 to 127 day*µg /mL, with two of three values achieving the target systemic exposure. Overall, tremelimumab concentrations were similar between disease cohorts in the dose-expansion phase; mean concentration-time profiles overlaid very well, with no notable differences in PK. Tremelimumab PK was similar between the dose-finding (D30+T1) and dose-expansion phases, regardless of whether the dose was or was not proceeded by a previous cycle of durvalumab.

An additional analysis was performed to explore the dose proportionality for durvalumab following a single infusion at 20 and 30 mg/kg using scatterplots of dose-normalized PK parameters (C_max_ and AUC from the time of dosing to the last measurable concentration) from patients enrolled in both the dose-finding and dose-expansion phases. Overall, the durvalumab exposure increased slightly more than proportionally to the increase in the dose. In the dose-finding phase, a 50% increase in the durvalumab dose resulted in a 260% to 270% increase in AUC_(0–28)_.

FIGURE S3 Geometric mean serum concentrations over time – dose-finding phase.

Only the first cycle of treatment is displayed. Time is presented with the day of dose administration assigned a value of 1 day. Bars indicate geometric standard deviation. Arm A, weight ≥35 kg; arm B, weight <35 kg.

**A. Durvalumab**

**
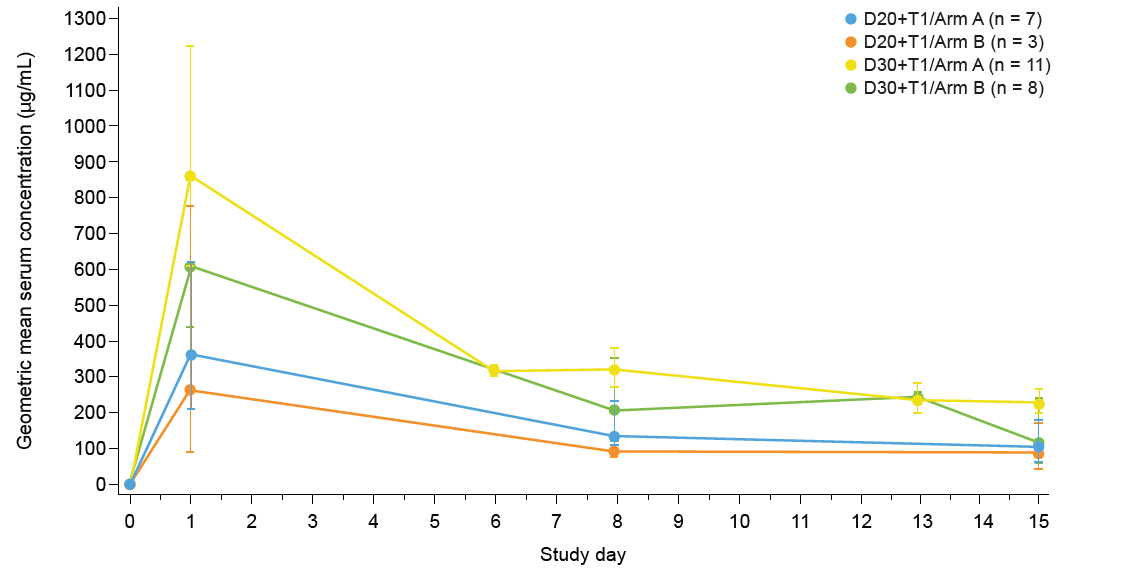
**

**B. Tremelimumab**

**
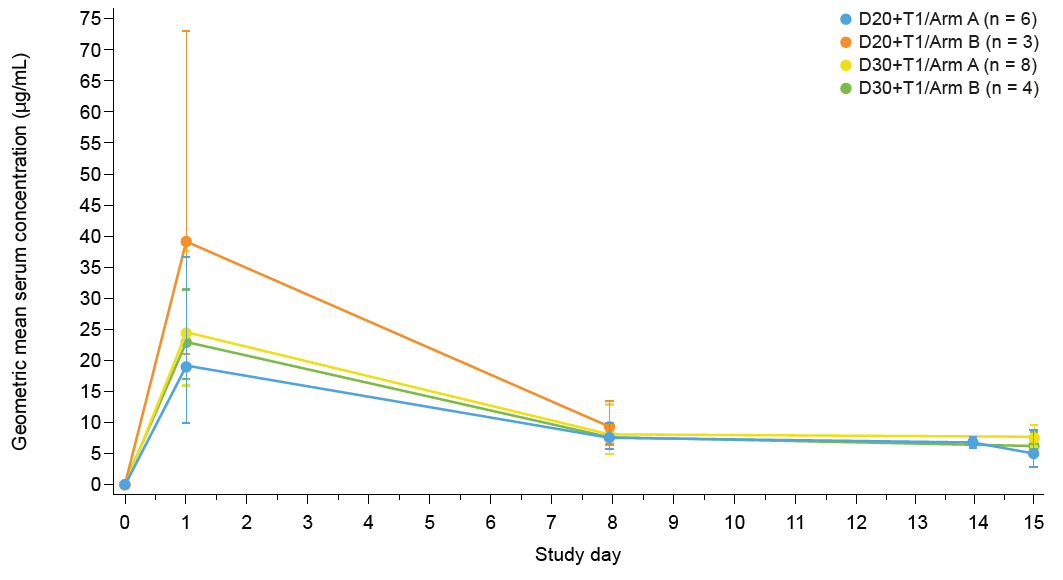
**

TABLE S4 Summary of PK parameters for durvalumab in the dose-finding phase.

| **PK parameter (units)** | **Statistic** | **Arm A: ≥35 kg** | | **Arm B: <35 kg** | |
| --- | --- | --- | --- | --- | --- |
|  |  | **D 20 mg/kg + T 1 mg/kg (n = 7)** | **D 30 mg/kg + T 1 mg/kg (n = 11)** | **D 20 mg/kg + T 1 mg/kg (n = 3)** | **D 30 mg/kg + T 1 mg/kg (n = 8)** |
| AUC_(0–14)_ (day*µg/mL) | Geomean (CV%)  Min–Max  n | 2650 (61.5)  1270–6790  7 | 5660 (17.7)  4620–8170  9 | 1830 (54.7)  1030–2760  3 | 3720 (46.9)  2280–7900  6 |
| AUC_(0–28)_ (day*µg/mL) | Geomean (CV%)  Min–Max  n | 3290 (50.1)  1650–6370  6 | 8790 (13.5)  7220–11400  8 | 2500 (55.4)  1420 – 3920  3 | 6380 (40.1)  4600–10700  4 |
| C_max_ (µg/mL) | Geomean (CV%)  Min–Max  n | 363 (58.0)  200–338  7 | 865 (36.0)  491–1440  11 | 275 (135)  88.6–632  3 | 612 (34.2)  415–1050  8 |
| C_min_ (µg/mL) | Geomean (CV%)  Min–Max  n | 48.6 (104)  11.9–135  6 | 169 (28.5)  114 – 295  8 | 21.7 (34.6)  16.1 – 31.3  3 | 118 (45.4)  73.4–176  4 |
| T_max_ (day) | Median  Min–Max  n | 0.094  0.09–0.10  7 | 0.087  0.00–0.14  11 | 0.088  0.09–6.94  3 | 0.087  0.08–0.09  8 |
| t_½λz_ (day) | Geomean (CV%)  Min–Max  n | 16.7 (47.1)  8.03–25.2  6 | 25.3 (56.5)  18.0–73.0  6 | 8.26 (NC)  8.26–8.26  1 | 15.6 (23.3)  13.2–18.3  2 |
| AUC_(0–14)_/d (day*µg/mL)/(mg/kg) | Geomean (CV%)  Min–Max  n | 132 (61.5)  63.7–340  7 | 189 (17.7)  154–272  9 | 91.6 (54.7)  51.6–138  3 | 124 (46.9)  76.0–263  6 |
| AUC_(0–28)_/d (day*µg/mL)/(mg/kg) | Geomean (CV%)  Min–Max  n | 164 (50.1)  82.5–318  6 | 293 (13.5)  241–382  8 | 125 (55.4)  71.0–196  3 | 213 (40.1)  153–358  4 |
| C_max_/d (µg/mL)/(mg/kg) | Geomean (CV%)  Min–Max  n | 18.1 (58.0)  10.0–45.3  7 | 28.8 (36.0)  16.4–47.9  11 | 13.7 (135)  4.43–31.6  3 | 20.4 (34.2)  13.8–35.1  8 |

Data represent single dose of durvalumab administered at cycle 1.

AUC_(0-t)_, area under the serum concentration-time curve from time zero to time ‘t’; C_max_, maximum serum concentration; C_min_, minimum serum concentration; CV, geometric coefficient of variance (%); d, dose administered; D, durvalumab; Geomean, geometric mean; Max, maximum; Min, minimum; n, number of patients included in analysis; NC, not calculable; PK, pharmacokinetic(s); t_½λz_, apparent terminal elimination half-life associated with the terminal slope (λz) of the semi-logarithmic concentration time curve, estimated as (ln2)/λz; T_max_, time to maximum serum concentration; T, tremelimumab.

**TABLE S5** Summary of PK parameters for tremelimumab in the dose-finding phase.

| **PK parameter (units)** | **Statistic** | **Arm A: ≥35 kg** | | **Arm B: <35 kg** | |
| --- | --- | --- | --- | --- | --- |
|  |  | **D 20 mg/kg + T 1 mg/kg (n = 7)** | **D 30 mg/kg + T 1 mg/kg (n = 11)** | **D 20 mg/kg + T 1 mg/kg (n = 3)** | **D 30 mg/kg + T 1 mg/kg (n = 8)** |
| AUC_(0–14)_ (day*µg/mL) | Geomean (CV%)  Min–Max  n | 127 (47.3)  58.2–181  5 | 160 (35.6)  90.0–259  7 | 165 (7.00)  157–173  2 | 149 (20.1)  122–191  4 |
| AUC_(0–28)_ (day*µg/mL) | Geomean (CV%)  Min–Max  n | 235 (11.2)  217–254  2 | 205 (22.9)  150–246  4 | NC | 208 (14.8)  178–239  3 |
| C_max_ (µg/mL) | Geomean (CV%)  Min–Max  n | 19.1 (73.3)  5.24–30.3  6 | 24.5 (44.7)  14.2–57.9  8 | 39.2 (68.8)  25.6–80.0  3 | 23.0 (31.7)  18.2–36.3  4 |
| C_min_ (µg/mL) | Geomean (CV%)  Min–Max  n | 3.71 (3.75)  3.61–3.81  2 | 3.45 (29.2)  2.42–4.85  4 | NC | 3.03 (50.3)  1.84–4.74  3 |
| T_max_ (day) | Median  Min–Max  n | 0.046  0.04–0.08  6 | 0.051  0.04–0.07  8 | 0.040  0.04–0.05  3 | 0.046  0.04–0.18  4 |
| t_½λz_ (day) | Geomean (CV%)  Min–Max  n | 16.8 (5.27)  16.2–17.5  2 | 18.7 (20.5)  14.8–21.4  3 | NC | 31.6 (86.0)  18.7–53.5  2 |
| AUC_(0–14)_/d (day*µg/mL)/(mg/kg) | Geomean (CV%)  Min–Max  n | 127 (47.3)  58.2–181  5 | 160 (35.6)  90.0–259  7 | 165 (7.00)  157–173  2 | 149 (20.1)  122–191  4 |
| AUC_(0–28)_/d (day*µg/mL)/(mg/kg) | Geomean (CV%)  Min–Max  n | 235 (11.2)  217–254  2 | 205 (22.9)  150–246  4 | NC | 208 (14.8)  178–239  3 |
| C_max_/d (µg/mL)/(mg/kg) | Geomean (CV%)  Min–Max  n | 19.1 (73.3)  5.24–30.3  6 | 24.5 (44.7)  14.2–57.9  8 | 39.2 (68.8)  25.6–80.0  3 | 23.0 (31.7)  18.2–36.3  4 |

Data represent single dose of tremelimumab administered (with durvalumab) at cycle 2.

AUC_(0-t)_, area under the serum concentration-time curve from time zero to time ‘t’; C_max_, maximum serum concentration; C_min_, minimum serum concentration; CV, geometric coefficient of variance (%); d, dose administered; D, durvalumab; Geomean, geometric mean; Max, maximum; Min, minimum; n, number of patients included in analysis; NC, not calculable; PK, pharmacokinetic(s); t_½λz_, apparent terminal elimination half-life associated with the terminal slope (λz) of the semi-logarithmic concentration time curve, estimated as (ln2)/λz; T_max_, time to maximum serum concentration; T, tremelimumab.

**FIGURE S4** Geometric mean serum concentrations over time – dose-expansion phase.

Only the first cycle of treatment is displayed. Time is presented with the day of dose administration assigned a value of 1 day. Bars indicate geometric standard deviation.

**A. Durvalumab**


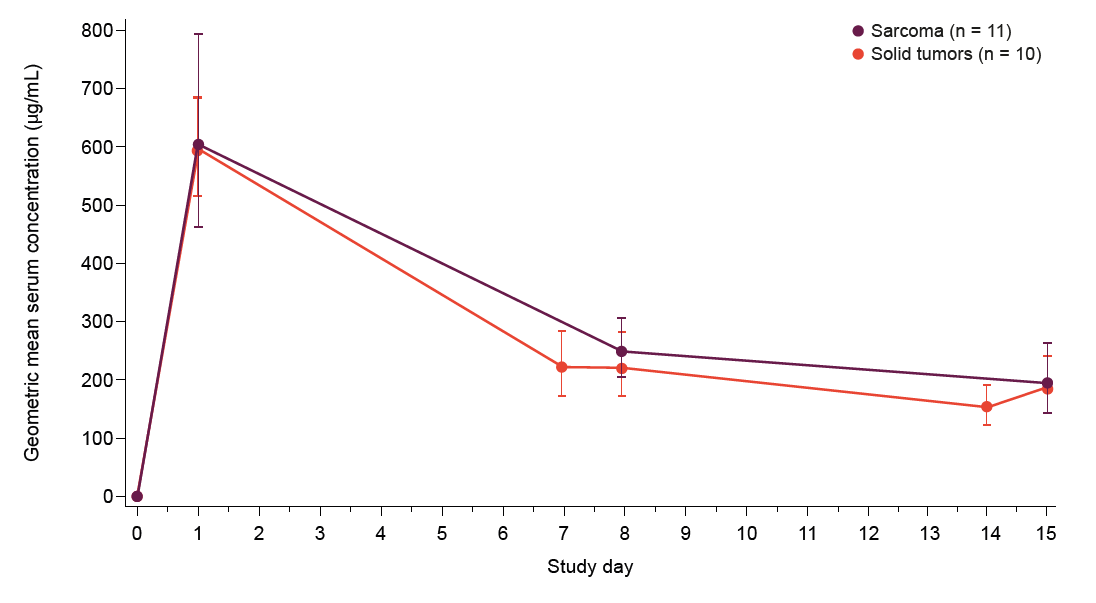


**B. Tremelimumab**


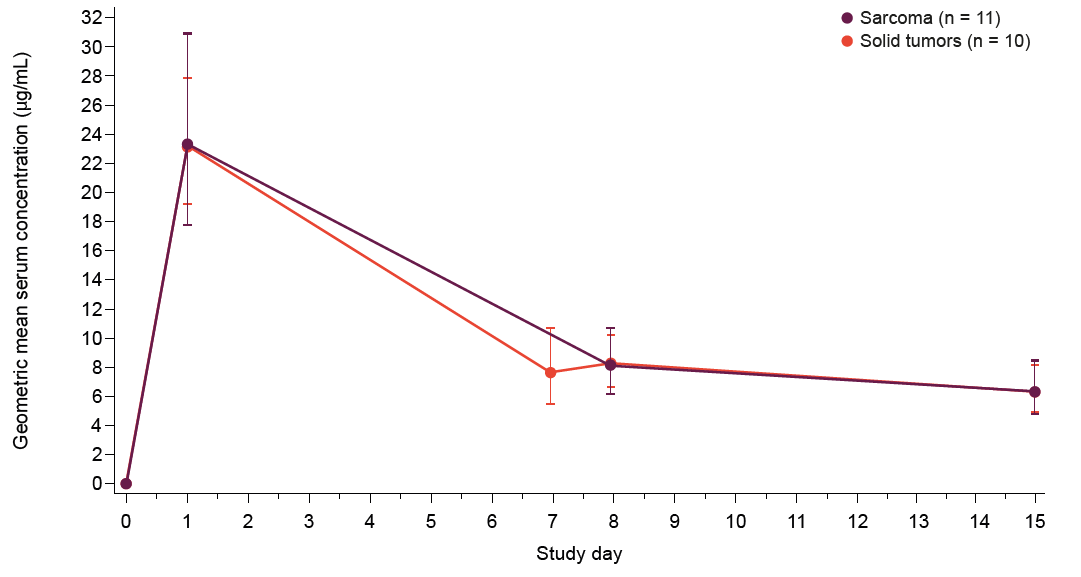


TABLE S6 Summary of PK parameters for durvalumab in the dose-expansion phase.

| **PK parameter (units)** | **Statistic** | **D 30 mg/kg + T 1 mg/kg**  **Sarcoma  (n = 11)** | **D 30 mg/kg + T 1 mg/kg Solid tumors  (n = 10)** |
| --- | --- | --- | --- |
| AUC_(0–14)_ (day*µg/mL) | Geomean (CV%)  Min–Max  n | 4240 (23.2)  3250–6820  10 | 3900 (20.0)  3030–5010  9 |
| AUC_(0–28)_ (day*µg/mL) | Geomean (CV%)  Min–Max  n | 6400 (23.7)  4560–10500  9 | 5880 (25.2)  3870–7520  5 |
| C_max_ (µg/mL) | Geomean (CV%)  Min–Max  n | 606 (27.7)  403–1090  11 | 595 (14.2)  447–706  10 |
| C_min_ (µg/mL) | Geomean (CV%)  Min–Max  n | 108 (29.6)  69.1–198  9 | 78.3 (94.1)  19.3–130  5 |
| T_max_ (day) | Median  Min–Max  n | 0.049  0.04–0.09  11 | 0.051  0.04–0.08  10 |
| t_½λz_ (day) | Geomean (CV%)  Min–Max  n | 17.4 (26.2)  12.5–25.2  8 | 14.2 (48.6)  6.67–23.0  5 |
| AUC_(0–14)_/d (day*µg/mL)/(mg/kg) | Geomean (CV%)  Min–Max  n | 141 (23.2)  108–227  10 | 130 (20.0)  101–167  9 |
| AUC_(0–28)_/d (day*µg/mL)/(mg/kg) | Geomean (CV%)  Min–Max  n | 213 (23.7)  152–349  9 | 196 (25.2)  129–251  5 |
| C_max_/d (µg/mL)/(mg/kg) | Geomean (CV%)  Min–Max  n | 20.2 (27.7)  13.4–36.3  11 | 19.8 (14.2)  447–706  10 |

Data represent single dose of durvalumab administered with tremelimumab at cycle 1.

AUC_(0-t)_, area under the serum concentration-time curve from time zero to time ‘t’; C_max_, maximum serum concentration; C_min_, minimum serum concentration; CV, geometric coefficient of variance (%); D, dose administered; d, durvalumab; Geomean, geometric mean; Max, maximum; Min, minimum; n, number of patients included in analysis; NC, not calculable; PK, pharmacokinetic(s); t_½λz_, apparent terminal elimination half-life associated with the terminal slope (λz) of the semi-logarithmic concentration time curve, estimated as (ln2)/λz; T_max_, time to maximum serum concentration; T, tremelimumab.

TABLE S7 Summary of PK parameters for tremelimumab in the dose-expansion phase.

| **PK parameter (units)** | **Statistic** | **D 30 mg/kg + T 1 mg/kg**  **Sarcoma  (n = 11)** | **D 30 mg/kg + T 1 mg/kg Solid tumors  (n = 10)** |
| --- | --- | --- | --- |
| AUC_(0–14)_ (day*µg/mL) | Geomean (CV%)  Min–Max  n | 183 (65.9)  112–892  10 | 150 (11.8)  126–173  8 |
| AUC_(0–28)_ (day*µg/mL) | Geomean (CV%)  Min–Max  n | 270 (58.1)  154–1000  9 | 228 (12.4)  184–252  6 |
| C_max_ (µg/mL) | Geomean (CV%)  Min–Max  n | 29.2 (86.0)  16.8–212  10 | 23.2 (18.8)  19.2–32.6  10 |
| C_min_ (µg/mL) | Geomean (CV%)  Min–Max  n | 3.91 (41.7)  2.05–6.92  9 | 3.40 (66.2)  1.03–5.45  6 |
| T_max_ (day) | Median  Min–Max  n | 0.049  0.04–0.06  10 | 0.052  0.04–0.07  10 |
| t_½λz_ (day) | Geomean (CV%)  Min–Max  n | 15.9 (26.2)  12.6–24.4  7 | 15.6 (40.4)  7.58–22.1  6 |
| AUC_(0–14)_/d (day*µg/mL)/(mg/kg) | Geomean (CV%)  Min–Max  n | 183 (65.9)  112–892  10 | 150 (11.8)  126–173  8 |
| AUC_(0–28)_/d (day*µg/mL)/(mg/kg) | Geomean (CV%)  Min–Max  n | 270 (58.1)  154–1000  9 | 228 (12.4)  184–252  6 |
| C_max_/d (µg/mL)/(mg/kg) | Geomean (CV%)  Min–Max  n | 29.2 (86.0)  16.8–212  10 | 23.2 (18.8)  19.2–32.6  10 |

Data represent single dose of tremelimumab administered with durvalumab at cycle 1.

AUC_(0-t)_, area under the serum concentration-time curve from time zero to time ‘t’; C_max_, maximum serum concentration; C_min_, minimum serum concentration; CV, geometric coefficient of variance (%); d, dose administered; D, durvalumab; Geomean, geometric mean; Max, maximum; Min, minimum; n, number of patients included in analysis; NC, not calculable; PK, pharmacokinetic(s); t_½λz_, apparent terminal elimination half-life associated with the terminal slope (λz) of the semi-logarithmic concentration time curve, estimated as (ln2)/λz; T_max_, time to maximum serum concentration; T, tremelimumab.

**TABLE S8.** Adverse events assessed by the investigator.

| **AE category, n (%)*** | **Dose-finding phase  (n = 29)** | | **Dose-expansion phase (n = 21)** |
| --- | --- | --- | --- |
|  | **D 20 mg/kg + T 1 mg/kg (n = 10)** | **D 30 mg/kg + T 1 mg/kg (n = 19)** | **D 30 mg/kg + T 1 mg/kg**  **(n = 21)** |
| Any AE | 9 (90.0) | 18 (94.7) | 19 (90.5) |
| Any AE possibly related to treatment | 5 (50.0) | 12 (63.2) | 16 (76.2) |
| Any AE possibly related to D only | 4 (40.0) | 10 (52.6) | 5 (23.8) |
| Any AE possibly related to T only | 0 | 0 | 0 |
| Any AE possibly related to D and T | 3 (30.0) | 8 (42.1) | 14 (66.7) |
| Any AE of grade 3 or 4 | 4 (40.0) | 6 (31.6) | 10 (47.6) |
| Any AE of grade 3 or 4 possibly related to treatment | 0 | 3 (15.8) | 4 (19.0) |
| Any AE of grade 3 or 4 possibly related to D only | 0 | 0 | 1 (4.8) |
| Any AE of grade 3 or 4 possibly related to T only | 0 | 0 | 0 |
| Any AE of grade 3 or 4 possibly related to D and T | 0 | 3 (15.8) | 3 (14.3) |
| Any serious AE | 1 (10.0) | 2 (10.5) | 9 (42.9) |
| Any serious AE possibly related to treatment | 0 | 2 (10.5) | 4 (19.0) |
| Any serious AE possibly related to D only | 0 | 0 | 2 (9.5) |
| Any serious AE possibly related to T only | 0 | 0 | 0 |
| Any serious AE possibly related to D and T | 0 | 2 (10.5) | 2 (9.5) |
| Any AE leading to discontinuation of D | 0 | 0 | 2 (9.5) |
| Any AE leading to discontinuation of T | 0 | 1 (5.3) | 1 (4.8) |
| Any AE leading to dose interruption | 0 | 2 (10.5) | 1 (4.8) |
| Any AESI | 2 (20.0) | 6 (31.6) | 10 (47.6) |
| Immune-mediated AEs | 0 | 1 (5.3) | 3 (14.3) |
| Grade 3 or 4 | 0 | 0 | 1 (4.8) |
| Infusion reaction AEs | 0 | 2 (10.5) | 1 (4.8) |

AE, adverse event; D, durvalumab; T, tremelimumab.

*Patients with multiple events in the same category were counted only once in that category. Patients with events in more than one category were counted once in each of those categories.

FIGURE S5 Percentage change from baseline in CD4+Ki67+ T cell absolute counts in evaluable patients.

**
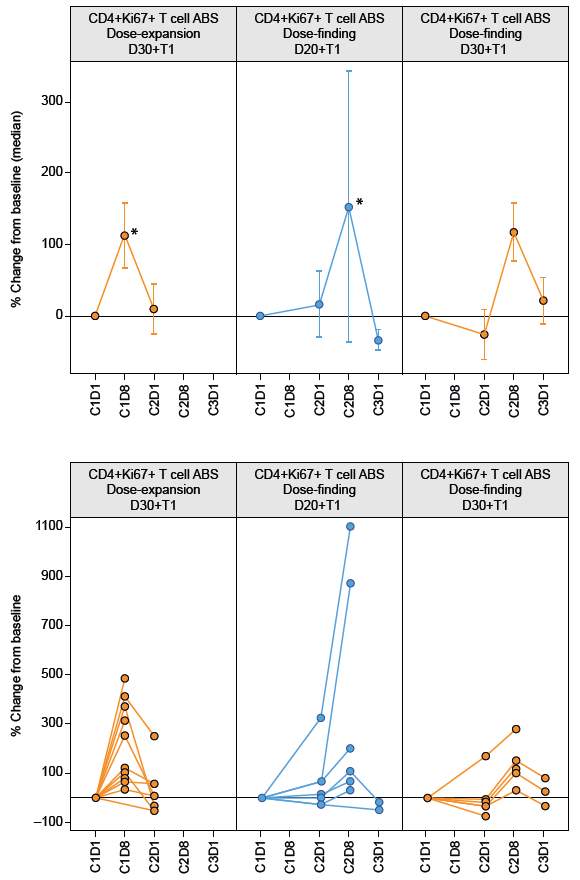
**

Tremelimumab (T) and durvalumab (D) doses in mg/kg and dosing schedule in the dose-finding and dose-expansion phases are indicated above the plots. Dose expansion was coadministration of both agents on cycle 1 day 1 (C1D1) following an every-4-week (Q4W) schedule. Dose finding was one Q4W cycle of D administration at the indicated dose followed by coadministration of both agents on cycle 2 day 1 (C2D1) following a Q4W schedule. Samples were collected prior to dosing on dose administration days. Black lines denote baseline value (0 change from baseline measurements). ABSs are reported in cells/mm^3^.

ABS, absolute count values; RV, range of variability.

*Significantly different median RV value (*P* < 0.05) by pairwise comparison using the Wilcoxon signed-rank test.

FIGURE S6 Percentage change from baseline in CD8+Ki67+ T cell absolute counts in evaluable patients.

**
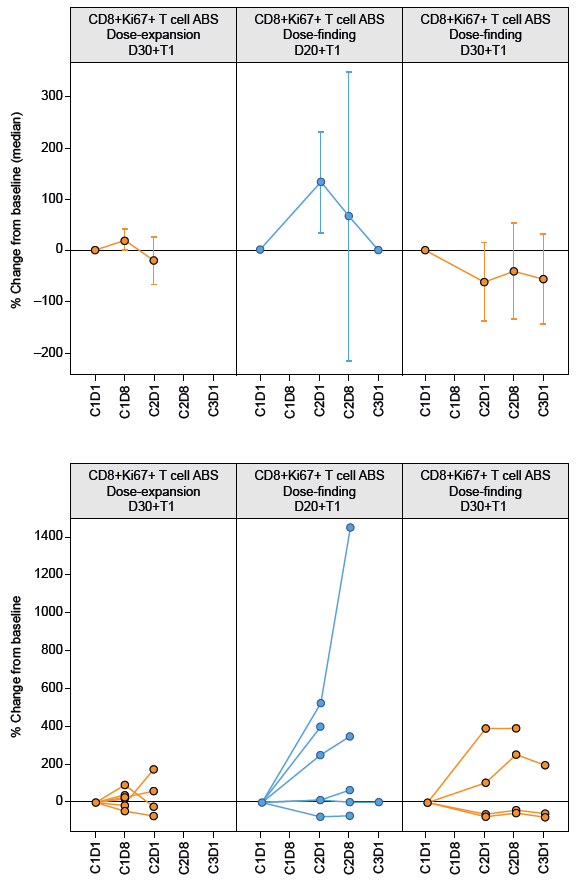
**

Tremelimumab (T) and durvalumab (D) doses in mg/kg and dosing schedule in the dose-finding and dose-expansion phases are indicated above the plots. Dose expansion was coadministration of both agents on cycle 1 day 1 (C1D1) following an every-4-week (Q4W) schedule. Dose finding was one Q4W cycle of D administration at the indicated dose followed by coadministration of both agents on cycle 2 day 1 (C2D1) following a Q4W schedule. Samples were collected prior to dosing on dose administration days. Black lines denote baseline value (0 change from baseline measurements). ABS are reported in cells/mm^3^.

ABS, absolute count values.

FIGURE S7 Percentage change from baseline in CD4+Ki67+ T cells (A) and CD8+Ki67+ T cells (B) in responding and nonresponding patients.

**A.**


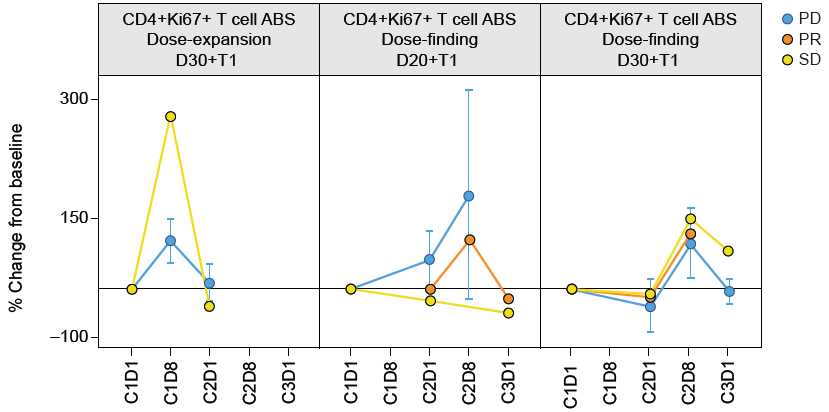


**B.**


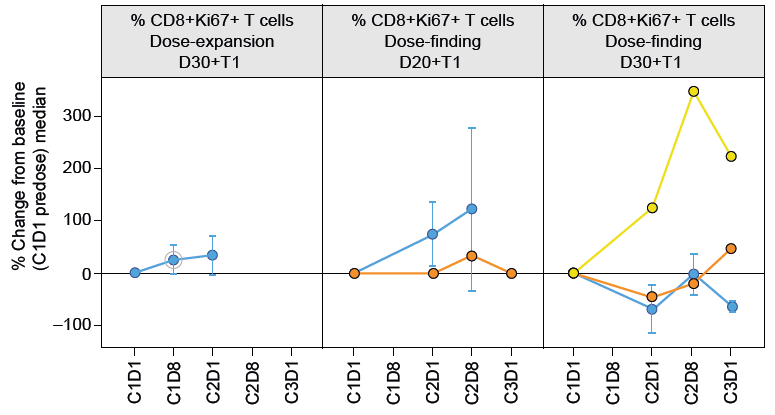


PD, progressive disease; PR, partial response; SD, stable disease.
